# A Systematic Review of Social Media Use to Discuss and View Deliberate Self-Harm Acts

**DOI:** 10.1371/journal.pone.0155813

**Published:** 2016-05-18

**Authors:** Michele P. Dyson, Lisa Hartling, Jocelyn Shulhan, Annabritt Chisholm, Andrea Milne, Purnima Sundar, Shannon D. Scott, Amanda S. Newton

**Affiliations:** 1 Alberta Research Centre for Health Evidence, Department of Pediatrics, University of Alberta, Edmonton, Alberta, Canada; 2 Ontario Centre of Excellence for Child and Youth Mental Health, Ottawa, Ontario, Canada; 3 Faculty of Nursing, University of Alberta, Edmonton, Alberta, Canada; 4 Department of Pediatrics, University of Alberta, Edmonton, Alberta, Canada; University of Stellenbosch, SOUTH AFRICA

## Abstract

**Objective:**

To conduct a systematic review of studies of social media platforms used by young people to discuss and view deliberate self-harm.

**Study Design:**

11 electronic databases were searched from January 2000 to January 2012 for primary research; in June 2014 an updated search of Medline was conducted. Grey literature sources were also searched. Search results were screened by two reviewers. Data were extracted by one reviewer and verified by another. Methodological quality was assessed using the Mixed Methods Appraisal Tool.

**Results:**

Due to heterogeneity in study objectives and outcomes, results were not pooled; a narrative analysis is presented. 26 studies were included. Most were conducted in Canada or the UK (30.8% each), used qualitative designs (42.3%), and evaluated discussion forums (73.1%). Participants were most often aged 19–21 years (69.2%), female (mean 68.6%), and 19.2% had a documented history of depression. The social media platforms evaluated were commonly supportive and provided a sense of community among users. Support included suggestions for formal treatment, advice on stopping self-harming behavior, and encouragement. Harms included normalizing and accepting self-harming behavior; discussion of motivation or triggers, concealment, suicidal ideation or plans; and live depictions of self-harm acts.

**Conclusions:**

Although this evidence is limited by its descriptive nature, studies identify beneficial and detrimental effects for young people using social media to discuss and view deliberate self-harm. The connections users make online may be valuable to explore for therapeutic benefit. Prospective, longitudinal investigations are needed to identify short- and long-term potential harms associated with use.

## Introduction

Worldwide, self-inflicted injury is the second leading cause of death for young people aged 15 to 19 years and tenth leading cause of death among 10 to 14 year olds.[1] Deliberate self-harm, a term used to describe non-fatal self-poisoning or self-inflicted injury irrespective of suicidal intent,[2] is under-recorded and can be a repetitive behavior.[3, 4] It is also estimated to be much more frequent than self-inflicted injury that results in death,[4] with a reported average lifetime prevalence ranging from 17% to 39% in adolescence and of 13.4% in early adulthood.[5–7]

Children and young people who engage in deliberate self-harm do not necessarily seek or receive care. Recent studies indicate between 5% and 25% of young people who report engaging in deliberate self-harm seek or receive healthcare before or after a self-harm event.[8–12] Young people have reported that help-seeking can be undermined by not knowing whom to ask for help and concern that their trust will be betrayed, as well as fear of causing more problems for themselves, being labeled as attention seeking, and hurting loved ones.[4, 11, 13] Studies have also found that healthcare professionals tend to have a negative view of people who self-harm,[14] and that when young people do disclose deliberate self-harm, despite significant difficulty in disclosure in many cases,[15] they often do not feel listened to.[16] Evidence from a recent systematic review indicates that young people with suicidal ideation or who deliberately self-harm are more likely to seek support from informal networks, most commonly consisting of their peers, than from healthcare professionals.[13]

In the context of young people placing a high value on peer-to-peer networks, accessing Internet resources, including those on social media, may play a significant role in how children and young people manage thoughts of deliberate self-harm and self-harm behaviors. Most young people in particular, spend a substantial amount of time online daily, and those who self-harm may access the Internet more frequently than those who do not self-harm.[17, 18] Inherent aspects of the Internet, and social media specifically, that may be appealing to young people who deliberately self-harm include the facilitation of information-seeking on a sensitive and stigmatized topic,[18] the potential for anonymity to ease communication of feelings and ideas that are difficult to convey in person,[19] and the creation of communities that bring together groups of individuals who are coping with similar problems.[20] While potential benefits of online platforms have been acknowledged, there is also concern that they may foster harmful actions, including triggering or encouraging the maintenance of self-harm behaviors.[21, 22]

Our objective was to conduct a systematic review of studies of social media platforms used by children and young people to discuss and view deliberate self-harm acts.

## Method

Our review methods were informed by our previous scoping work examining the uses of social media in healthcare.[23] From these results, we identified pediatric mental health as a priority area for further study. The protocol is available upon request from the authors.

### Search Strategy

A research librarian searched 11 databases from January 1, 2000 to January 13, 2012: Medline, CENTRAL, ERIC, PubMed, CINAHL, Academic Search Complete, Alt Health Watch, Health Source, Communication and Mass Media Complete, Web of Knowledge, and ProQuest Dissertation and Theses Database. No study design restrictions were applied to the search. Grey literature was searched, including key organizations with an interest in social media (Pew Internet and American Life Project, Cybercitizen Health, the Centers for Disease Control and Prevention, and the Mayo Clinic Center for Social Media), ClinicalTrials.gov, key journals, and conference proceedings; reference lists of relevant and included studies were checked. The complete search was updated in May 2013 (up to April 24, 2013); another update was conducted in Medline, our principal database, to June 24, 2014 to identify highly relevant studies published between 2013 and 2014. The search strategy for Medline is available online (see S1 Appendix).

### Study Selection

Two independent reviewers screened titles and abstracts for eligibility. The full text of studies assessed as ‘relevant’ or ‘unclear’ was independently evaluated by two reviewers using a standardized form. Discrepancies were resolved through adjudication by a third party.

Studies were included if they reported primary research that described or evaluated children or young people’s use of social media platforms related to self-harm or suicidality. Analytic quantitative designs were included to assess the impact of social media use, and descriptive and qualitative designs were included to provide context. We defined social media as: collaborative projects, blogs or microblogs, content communities, social networking sites, and virtual worlds.[24] To maintain a focus on similar technological platforms, we excluded studies that evaluated mobile technologies (e.g., text messaging) and real-time exchanges facilitated by technology (e.g., Skype). Outcomes were not defined *a priori* as they were to be incorporated into our description of the field. Only English-language studies were included.

### Data Extraction and Quality Assessment

Data were extracted into standardized forms (Microsoft Excel; Microsoft, Redmond, Washington, USA) by one reviewer, and were verified for accuracy and completeness by another; discrepancies were resolved through consensus. Data were extracted on study and population characteristics, description of the social media tools used, outcomes measured, results, and authors’ conclusions.[25]

Quality assessment of included studies was conducted using the Mixed Methods Appraisal Tool (MMAT),[26, 27] which includes specific criteria for qualitative, mixed methods, and quantitative studies (see S2 Appendix, available online). Two reviewers independently applied the MMAT to each study and resolved disagreements through discussion.

### Data Synthesis and Analysis

Due to heterogeneity in study objectives and outcomes, we did not pool study findings; we described the results narratively, stratified by quality assessment score, and in evidence tables. Descriptive statistics were calculated using StataIC 11 (StataCorp, College Station, Texas, USA). Qualitative data were analyzed using content analysis techniques. Our analysis emphasizes studies scoring 75% or higher on the MMAT (meeting at least 3 of 4 criteria; ‘high quality’ studies), with lower quality studies used to provide additional context for the results: reinforcing findings or identifying gaps or discrepancies.

## Results

### Description of Included Studies

**Fig 1:**
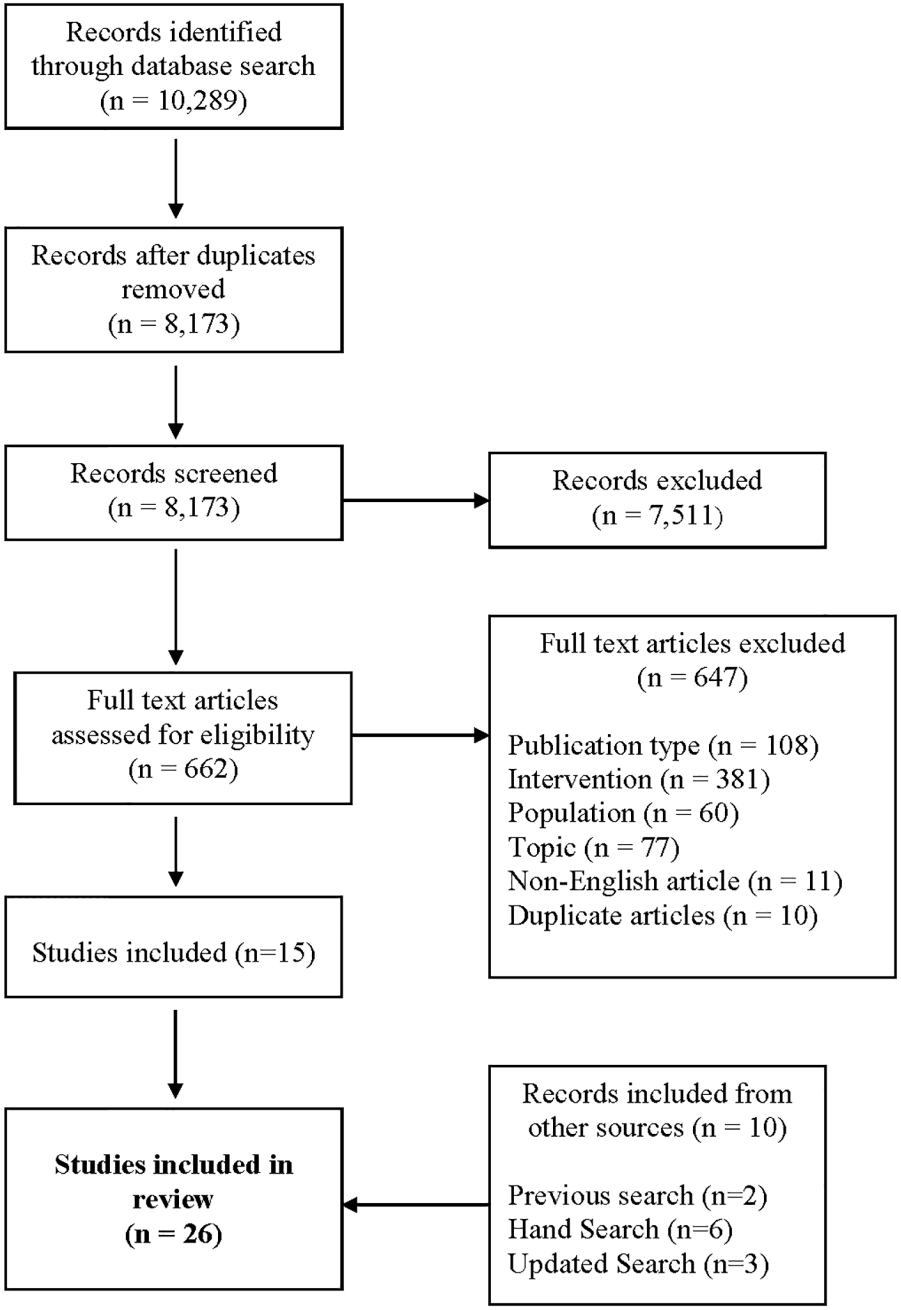
Flow Diagram of Study Selection. Details of the flow of information through the phases of the systematic review.

We screened 8,173 records and included 26 publications that met our criteria (Fig 1, S3 Appendix). Table 1 provides an overall description of the included studies. The majority of the included studies were conducted in Canada and the United Kingdom (30.8% each), were descriptive studies using content analysis or were qualitative designs (34.6% and 42.3%, respectively), and evaluated discussion forums (73.1%). Most discussion forums (68.4%) were user-initiated, although a smaller proportion were driven and moderated by professionals. Participants were most often aged 19–21 years when age was reported (69.2%), female (mean 68.6%), and many had a documented history of depression (19.2%).

**Table 1:** Description of included studies (n = 26). Figures are number, percentage unless otherwise stated.

**Study Characteristics**	**n**	**%**
Country of corresponding author		
Canada	8	30
Israel	1	4
Germany	1	4
Hungary	1	4
Japan	2	8
Sweden	1	4
UK	8	31
USA	4	15
Start date, median year (range)	2008 (1994–2010)	
Duration in days / months, median (range)	3 months (1 day– 11 months)	
Sample size analyzed, median (range)	Participants: 100 (10–9,990)	
	Online posts: 77 (8–26,100)	
Design		
Cross Sectional	5	19
Descriptive	9	35
Qualitative	11	42
Mixed Methods	1	4
Setting^a^		
University / College	1	4
Online Community	26	100
Social media platform^b^		
Blog	2	8
Bulletin board / discussion forum	19	73
Facebook	2	8
Myspace	2	8
YouTube	4	15
Mixi	1	4
**Study Population Characteristics**	**n**	**%**
Gender, mean % (female / male)	69 / 27	
Age		
12–18 years	15	58
19–21 years	18	69
Not reported	8	31
Ethnicity, mean %		
White	84	
Black	9	
Latino / Hispanic	5	
Other / Not reported	9	
Pre-existing mental health conditions		
Anxiety / anxiety disorders	3	12
Bipolar disorder	2	8
Depression	5	19
Eating disorder	1	4
Obsessive compulsive disorder	1	4
Panic disorder	1	4
Post-traumatic stress disorder	1	4
Substance use / abuse	2	8

^a^ Percentages do not add up to 100% because some studies were conducted in multiple locations.

^b^ Percentages do not add up to 100% because some studies evaluated multiple social media platforms.

### Methodological Quality of Included Studies

Methodological quality was variable across the included studies (see S4 Appendix). Scores ranged from 25.0% (1 of 4 criteria met) to 100% (4 of 4 criteria met). Of the five cross-sectional studies, one met all four criteria,[28] and the remaining studies scored 50.0% or lower.[29–32] The qualitative studies were the highest quality, with 10 of 11 scoring 75.0% or higher.[19, 21, 33–40] Descriptive studies using content analysis demonstrated considerable variation, with three scoring 50.0% or lower,[41–43] and six scoring 75.0% or higher.[18, 44–47]

### Use of Social Media Platforms to Discuss and View Deliberate Self-Harm Acts

Nine high quality studies provided a general description of how social media is used by children and young people who self-harm (Table 2).[18–20, 37–40, 45, 46] The platforms were often described as supportive, and participants provided each other with encouragement and empathy.[20, 38–40, 46] A strong sense of community developed within the online groups.[20, 33, 34, 36, 40] Users were able to cultivate a sense of belonging in an understanding and validating environment. In this community, users became ‘insiders,’ able to share their experiences with supportive people having similar perspectives.[34] Support was common, tending to be emotion-focused, rather than problem-focused.[20] Where problems were presented, this was often used as a strategy for the poster to initiate more detailed personal narratives.[38] While support was freely provided, there was also an expectation of reciprocity between members.[38] The communities were associated with a sense of not wanting to let the other members down by not posting, or by relapsing into self-harming behavior after being encouraged not to.[36] However, despite creating positive community ties, the groups evaluated tended to resist making close personal connections, remaining guarded in their interactions.[35, 41]

**Table 2:** Characteristics of studies reporting features of social media platforms used to discuss and view deliberate self-harm acts.

Author, Publication Date (Country)	Study Design Features	Study Population	Social Media Platform	Key Findings	MMAT Scoring
Cash, 2013[19] (USA)	Qualitative analysis of online profiles and comments to identify themes related to relationships, mental health and substance use, and method of deliberate self-harm	1,038 Myspace posts regarding deliberate self-harm	Myspace	- Comments referenced a significant amount of hopelessness, despair and desperation	100
				- Public websites were used to reveal suicidal thoughts, behaviors, and possible intent	
Smithson, 2011[38] (UK)	Qualitative analysis of online forum posts to identify site norms and expectations. Posts by individuals who deliberately self-harm and use self-harm discussion forums^a^		Discussion forum (SharpTalk)^b^	- Site norms and expectations were largely created by the participants	100
				- Requests for advice were often followed by a request for emotional support or empathy	
				- Self-harm was normalized, and users combined perceptions of deliberate self-harm as a routine activity with suggestions on how to be ‘safe’	
Rodham, 2007[20] (UK)	Qualitative analysis of online forum posts to evaluate users’ interactions and identify how they established the function of the message board	65 posts by users of deliberate self-harm discussion forums	Discussion forum	- Individuals using the message boards considered them to be a significant source of support	100
				- Responses to posts describing harmful behaviors tended to minimize the severity of and normalize self-harm actions	
Fekete, 2002[46] (Australia, Canada, France, Israel, Japan, the Netherlands, UK, USA)	Descriptive analysis of forum posts to identify the most frequent topics	382 posts to a suicide-based self-help group	Discussion forum	The most common topics of discussion identified included: asking for and providing support; suicide models, pacts and imitation; suicide methods and information; consequences of suicide	100
Lewis, 2014[18] (Canada)	Descriptive analysis of health information website data to identify the quality of information regarding deliberate self-harm	340 websites (including blogs) used by individuals who deliberately self-harm	Blog	Online searches for information on deliberate self-harm were frequent, and often resulted in non-credible and low-quality information containing common myths	75
Duggan, 2012[45] (Canada)	Descriptive analysis of discussion forums, social media groups, and YouTube videos to identify the scope of deliberate self-harm activities reported online	20 online platforms used by individuals who deliberately self-harm	Blog, discussion forums, Facebook, Myspace, YouTube	- Peer-led, informal discussion forums were accessed more often than professional forums and contained content that could prompt deliberate self-harm	75
				- Deliberate self-harm is well represented on social networking sites and YouTube	
Sharkey, 2012[40] (UK)	Qualitative analysis of online forum posts to identify the nature of online interactions	77 posts by individuals who deliberately self-harm and use self-harm discussion forums	Discussion forum (SharpTalk)^b^	- Participants demonstrated endearments, encouragement, and solidarity	75
				- Mitigating devices such as indirectness, disclaimers, and hedges, were frequently used to reduce the impact of ‘threatening’ interactions	
Horne, 2009[37] (USA)	Qualitative analysis of online forum posts to identify the format and frequency of the initial posts in a discussion thread	329 posts on suicide discussion boards by users who reported being suicidal or having suicidal feelings	Discussion forum	- The function of the forum was partly to allow individuals to experiment with suicidal identities	75
				- Users demonstrated their authenticity through four techniques: providing detailed personal narratives, presenting themselves as beyond depression, making a rational case for suicide, and not explicitly asking for help	
Adler, 2008[39] (UK)	Qualitative analysis of online forum posts and individuals to identify facets of cyber community use	81 individuals who deliberately self-harm and are users of self-harm discussion boards and online communities	Discussion forum	- Community size, demographics, level of activity, and orientation were considerations in selecting an online community	75
				- Online communities varied in level of regulation, focus, and stance on self-harming behaviors	
				- Finding a community gave participants a sense of identity	
				- Participation in an online community was situational, with users coming and going as they needed support	

^a^ Sample size not reported

^b^ Professionally-driven forum

A prominent group norm included a high value placed on ‘authenticity.’[37, 38, 48] Acceptance by the group required that users’ self-harming behavior was perceived as the result of ‘real’ problems, rather than to garner attention.[48] Users often acknowledged, but rejected, advice provided by other members of the group, potentially suggesting that if their problem was easy to solve, they would be ‘inauthentic.’[38] Interactions between participants were often guarded and emphasized their vulnerability as a potential strategy to avoid threatening exchanges.[40]

Two studies assessed references to mental illness or overt suicidal content (ideation or behavior) posted on social media.[19, 45] In one study of content posted on Facebook, Myspace, YouTube, and discussion boards (five websites per platform), all of the discussion boards and 80% of YouTube videos sampled contained information related to co-occurring mental illnesses, and 100% of discussion forums and 40% of YouTube videos contained explicit references to suicidal thinking or behavior.[45] Facebook and Myspace profiles often contained content that was linked to mental illness. The second study examined Myspace comments indicating potential suicidality.[19] Among these posts, 51.6% did not provide a specific context to describe the situation or rationale, only the desire to die. However, included in these comments were some indications of previous attempts and/or ideation.

### Potential Benefits Associated with the Use of Social Media to View and Discuss Deliberate Self-Harm Acts

Membership in social media-based groups was associated with benefits for participants, and these were evaluated in five methodologically rigorous studies (Table 3).[34–36, 44, 47] The relationships developed with other members provided a sense of purpose, being understood, and acceptance.[31, 36, 44] Users seeking support often transitioned into becoming providers of support, which was associated with an increased sense of competence and usefulness.[36, 44, 49] In one study, this was described as providing users with two positive identities: as the ‘understander,’ one is perceived as thoughtful, compassionate, and helpful; and as the one ‘understood,’ the person is supported, cared for, and accepted.[34] Support came in the form of suggestions of formal treatment, advice on stopping self-harming behavior or on harm reduction, and delivery of encouraging comments.[33, 49] In some cases, the sites were used as an alternative to self-harming behavior.[34] Two further studies also suggested benefits. One study of a professionally-driven discussion forum evaluated the correlation between number of posts and distress over time and found that higher use of the forum during the first month was associated with lower levels of distress in the second and third months.[43] None of the other social media platforms created or moderated by professionals evaluated a measure of therapeutic effectiveness.[32, 36, 38, 40, 44] In one study, 41.8% of respondents indicated that membership in an online group had reduced their self-harming behavior.[31]

**Table 3:** Potential benefits reported in primarily positive studies regarding the use of social media platforms to discuss and view deliberate self-harm acts.

Author, Publication Date (Country)	Study Design Features	Study Population	Social Media Platform	Key Findings	MMAT Scoring
Smithson, 2011[36] (UK)	Qualitative analysis of online forum posts to identify how individuals become members of online discussion communities	77 posts by users of discussion forums who deliberately self-harm	Discussion forum (SharpTalk)^a^	- Users felt an obligation to not let others down by engaging in self-harm	100
				- Users felt accepted and a sense of belonging	
				- Forum members were more likely than forum moderators to address perceived deviance in posts and to provide healthcare advice	
Baker, 2008[34] (UK)	Qualitative analysis of semi-structured interviews to understand empathetic understanding among users of online self-harm discussion forums	10 users of deliberate self-harm discussion forums	Discussion forum	- Users found empathetic understanding in the online community	100
				- Online membership afforded a positive identity	
				- Some members used forums as an alternative to deliberate self-harm	
Miller, 1998[35] (USA)	Qualitative analysis of online forum posts to identify help-seeking interchanges	98 users of suicide discussion forums	Discussion forum	- Online exchanges between community members may be superior to psychotherapeutic practices in some instances because they are richer and virtually open-ended	100
Lewis, 2011[47] (Canada)	Descriptive analysis of posts from personally constructed websites on deliberate self-harm	71 self-harm discussion forums used by individuals who deliberately self-harm	Discussion forum	- Websites often contained supportive messages for those who deliberately self-harm	75
Greidanus, 2010[44] (Canada)	Descriptive analysis of online forum posts to identify member experiences and purposes for seeking help	10 message threads on a discussion forum from users who reported being suicidal	Discussion forum^a^	- Help-seekers felt their experiences were understood and shared by other members	75
				- With time, help-seekers often progressed to providing others with feedback and support	

^a^ Professionally-driven forum

### Potential Harms Associated with the Use of Social Media to View and Discuss Deliberate Self-Harm Acts

Four high quality studies focused on the potential harms of social media use in relation to acts of deliberate self-harm (Table 4).[21, 28, 33, 47] One cross-sectional study of 9,990 members of online suicidal ideation communities found that the likelihood of suicidal ideation increased with an increase in the number of communities the user belonged to, the proportion of suicidal neighbors in their social network, and the degree of social isolation.[28]

**Table 4:** Potential harms reported in primarily negative studies regarding the use of social media platforms to discuss and view deliberate self-harm acts.

Author, Publication Date (Country)	Study Design Features	Study Population	Social Media Platform	Key Findings	MMAT Scoring
Masuda, 2013[28] (Japan)	Cross-sectional analysis of online posts to identify factors contributing to membership in suicidal ideation communities	9,990 online members of suicidal ideation communities	Mixi	- Suicidal ideation was more likely when users belonged to more communities, had a higher proportion of suicidal neighbors in their social network, and were socially isolated	100
				- Age, gender, number of friends contributed little to suicidal ideation	
Lewis, 2012[21] (Canada)	Qualitative analysis of online responses to the most viewed YouTube videos of deliberate self-harm	22,311 comments made by viewers of deliberate self-harm videos	YouTube	- Sharing personal experiences was a strong motivator for video viewing	100
				- Responses rarely encouraged or mentioned recovery, and often expressed admiration and validation for the videos, their messages, and the poster, potentially maintaining self-harming behavior	
Niwa, 2012[33] (Australia, Canada, Middle East, Europe, UK, USA)	Qualitative analysis to identify themes in online postings	998 online posts from participants across four deliberate self-harm Facebook groups	Facebook	- Exposure to trolling and flaming comments was harmful, leading to a high proportion of antagonistic and defensive posts	100
Lewis, 2011[47] (Canada)	Descriptive analysis of posts from personally constructed websites on deliberate self-harm	71 self-harm discussion forums used by individuals who deliberately self-harm	Discussion forum	- Risks associated with the websites included behavior normalization, reinforcement, and triggering of the behavior, as well as learning additional methods to self-harm	75

While the provision of support through social media platforms was common, potential dangers of these sites included creating groups that resulted in further isolation from the rest of society,[34] and minimizing the severity of self-harming situations by focusing on emotional support, normalizing and accepting the behavior at the expense of offering alternative coping strategies.[20, 38] Other themes common to the posts included discussion of motivation or triggers (6.1% to 19.5%),[33, 49] concealment (2.9% to 50.7%),[33, 46, 47, 49] and suicidal ideation (5.0%)[33] or plans (26.2%).[37]

Live depictions of deliberate self-harm included partial asphyxiation[41] and cutting.[42] In both cases, the self-harming behaviors were posted as videos on YouTube. In the study of partial asphyxiation, 41.0% to 88.0% of videos, depending on the method of asphyxiation used, showed actions resulting in seizures;[41] of the videos of cutting, 22.0% were coded as low severity (superficial cuts), 27.0% were moderate (cuts with obvious blood flowing), and 13.0% were high (gushing blood or a gaping wound).[42] Negative exchanges captured on the sites included ‘trolling’ (deliberate provocation) and ‘flaming’ (mocking or encouraging self-harm; 21.6% of posts in one study),[33] making suicide pacts and posting models to follow (7.5%),[46] and seeking information on suicide methods (7.0%).[46] In one study, 11.0% of respondents reported that belonging to an online self-harm group had a negative impact on their self-harming behavior (i.e., triggered self-harm).[31]

## Discussion

The perception around social media use in this population is commonly centered on its potential and actual harms, which receive media attention and cause concern among parents. In our systematic review, we identified 26 studies that evaluated social media use by children and young people to view and discuss deliberate self-harm, and found preliminary evidence to suggest that harms do exist, but there are also benefits associated with these platforms. These studies outlined how social media forums can provide an informal support network, while also encompassing the risk of triggering self-harmful behavior.

In our review, the identification of existing benefits suggests the potential for harnessing the positive effects of social media in understanding children and young people who self-harm, with the aim of developing acceptable intervention and prevention strategies.[4] The primary theme to emerge from the data regarding the potential benefits of social media use was that it could create a sense of community, allowing users to feel supported and accepted. This connectedness with peers is a critical part of adolescent life,[50] and perhaps particularly so among individuals who feel vulnerable, misunderstood, and stigmatized. In many cases, children and young people who deliberately self-harm choose not to engage with health services, precluding traditional means of treatment and support. However, online communities formed via social media platforms may be sought out as alternate avenues for support. This is likely a valuable avenue for exploration in a group that is resistant to traditional help-seeking,[13] especially if measures are taken to educate clinicians, parents, and youth on strategies to minimize harms. Informal support networks may also provide opportunities to reach out to young people to decrease stigma and promote meaningful engagement with professional sources of help.[4, 51] While the current evidence suggests that there are perceived benefits of belonging to social media communities among users, future prospective research should evaluate whether there are improved health outcomes over time.

Of concern for children and young people who use social media to discuss and view deliberate self-harm acts is the potential for a ‘normalizing effect.’ That is, the use of social media communities may affect a child/young person’s perceptions of deliberate self-harm and lead to the sustained adoption of maladaptive coping behaviors and the potential for triggering future acts of self-harm. The content of many of social media platforms also directly violates recommendations for responsible reporting of self-harm and suicide in the media, which are in place to minimize the potential negative impact on future actions, such as copycats, social contagion, and suicide clusters. Guidance from national bodies in North America and the United Kingdom advise against providing details of the method, publishing photos, reporting romanticized reasons for the acts, and expressing approval for the behavior;[52–54] however, these practices were common on the social media platforms described in our review. Major social media platforms, including Tumblr, Pinterest, Instagram, and Facebook, have implemented content policies in which posts related to self-harm are banned, unsearchable, or trigger public service announcements or links to counseling and prevention resources.[55–59] Despite these measures, communities of users continue to find ways to circumvent the restrictions, and not all platforms monitor content.[51, 55] While the open nature of social media poses significant challenges for ensuring high-quality and responsible content, where possible, sites such as forums, especially when intended to be therapeutic, should be professionally moderated and monitored for guideline adherence. Based on the findings from this review, there may be merit in expanding current media recommendations to include social media platforms and target platform developers/owners to regulate their forums. Given the challenges in moderating these forums, however, this review also points to the need to develop guidance for parents and young people outlining relevant considerations to ensure safe Internet use, as well as appropriate steps to take to monitor social media behavior.

We identified several studies of platforms that were developed by healthcare professionals or researchers,[32, 36, 38, 40, 43, 44] but only one that focused on therapeutic benefit (emotional relief) and included a measure of effectiveness.[43] As most of these evaluations provided narrative descriptions of how users engaged with the social media platforms, important avenues for future research will include using more robust prospective study designs to address implications for health outcomes, including issues such as therapeutic benefits, unintended harms, and differences in use and effectiveness between professionally-driven and user-initiated sites.

The body of literature to date has been descriptive, and as such, limited conclusions can be drawn regarding the overall impact of social media in the population. We emphasized the findings of the most methodologically rigorous studies identified in our review; however, none provided longitudinal evaluations of the impact of social media use on a user’s deliberate self-harming behaviors or other critical outcomes such as death by suicide. While these descriptive studies have suggested associations between use of social media platforms and both benefits and harms, rigorous evaluations specifically designed to address questions about long-term impact are needed. We were also limited by the descriptions of the populations used in the primary studies, which encompassed both suicidal and non-suicidal behaviors, potentially restricting conclusions that can be made with respect to the intent underlying acts of deliberate self-harm. Users’ age was often indeterminable from the studies, and when it was reported, it covered a wide range. Different age groups use the Internet and social media differently; therefore, future research should delineate usage patterns. It will also be important to identify whether use of certain platforms is associated with more or less benefit or harm, given that our conclusions are based on a sample of studies predominantly evaluating discussion forums. Moving forward, the development of a common nomenclature for both self-harm terminology and Internet activities will advance future generation of sound data on important subgroups. Lacking robust evidence demonstrating a relationship between social media use and self-harming behaviors, we recommend that clinicians be mindful of general Internet safety practices[60] and the main tenets of media literacy, including the construction, understanding, interpretation, and implications of messages.[61]

## Conclusions

Although this evidence is limited by its descriptive nature, studies identify beneficial and detrimental effects for young people using social media to discuss and view deliberate self-harm. The connections users make online may be valuable to explore for therapeutic benefit. Prospective, longitudinal investigations are needed to identify short- and long-term potential harms associated with use.

## Supporting Information

S1 Appendix Medline Search Strategy. (DOCX)

S2 Appendix Mixed Methods Appraisal Tool Criteria. (DOCX)

S3 Appendix List of Excluded Studies. (DOCX)

S4 Appendix Mixed Methods Appraisal Tool Scoring of Included Studies. (DOCX)

S1 Table PRISMA Checklist. (DOCX)
