# Supplementary material for: A Systematic Review of Social Media Use to Discuss and View Deliberate Self-Harm Acts
Source: PLoS One. 2016 May 18;11(5):e0155813. doi: 10.1371/journal.pone.0155813 (PMC4871432; doi:10.1371/journal.pone.0155813)
Supplement: S1 Appendix — (DOCX) [file pone.0155813.s001.docx]

**S1 Appendix. Medline Search Strategy**

Database: Medline via Ovid <1946 to Present>

Date Searched: 24 April 2014

| **Medline search strategy** |
| --- |
| **Internet and social media related MeSH:**  1. Computer-Assisted Instruction/  2. Computers/td, ut  3. Electronic Mail/  4. Mass Media/td, ut  5. Medical Informatics/  6. Online Systems/td, ut  7. Search Engine/  8. User-Computer Interface/  9. exp Internet/ |
| **Internet and social medial related keywords:**  10. blog*.mp.  11. e-health.mp.  12. Facebook*.mp.  13. (forum* adj3 (internet or web* or chat*)).mp.  14. Googl*.mp.  15. "Health 2.0".mp.  16. "Medicine 2.0".mp.  17. microblog*.mp.  18. myspace.mp.  19. (online or on-line).mp.  20. PatientsLikeMe.mp.  21. podcast*.mp.  22. Second Life.mp.  23. (social adj3 media*).mp.  24. (Social adj3 network*).mp.  25. (twitter or tweet*).mp.  26. user generated content.mp.  27. (virtual adj3 (world* or communit*)).mp.  28. ("Web 2.0" or "Web 2").mp.  29. web-based.mp.  30. WebMD.mp.  31. (website* or web site* or webpage* or web page*).mp.  32. wiki*.mp.  33. World Wide Web.mp.  34. YouTube.mp. |
| 35. **or/1-34** [Internet/social media MeSH and keywords] (135,642) |
| **Mental Health MeSH Terms:**  36. Anorexia Nervosa/  37. Bulimia Nervosa/  38. Eating Disorders/  39. Depressive Disorder, Major/  40. Impulsive Behavior/  41. Mental Disorders/  42. Mental Health/  43. Personality Disorders/  44. Psychotic Disorders/  45. Risk taking/  46. Schizophrenia, Childhood/  47. exp Anxiety Disorders/  48. exp "Attention Deficit and Disruptive Behavior Disorders"/  49. exp Bipolar Disorder/  50. exp Mood Disorders/  51. exp Schizophrenia/  52. exp Self-Injurious Behavior/  53. exp Substance-Related Disorders/ |
| **Mental Health keyword terms:**  54. (anorexia or bulimia).tw.  55. (anxiety adj1 (disorder* or behavio?r* or syndrom*)).tw.  56. ((bipolar or cyclothymic) adj1 disorder*).tw.  57. ((choking or asphyx*) adj1 game*).tw.  58. (depress* adj1 (disorder* or behavio?r* or syndrom*)).tw.  59. (eat* adj1 disorder*).tw.  60. (hyperkinetic adj1 (disorder* or syndrom*)).tw.  61. (mood adj1 (disorder* or behavio?r* or syndrom*)).tw.  62. (("obsessive compulsive" adj1 (disorder* or behavio?r* or syndrom*)) or OCD).tw.  63. (personalit* adj1 (disorder* or syndrom*)).tw.  64. (psychotic adj1 (disorder* or behavio?r* or syndrom*)).tw.  65. (risk* adj1 behavio?r*).tw.  66. (self adj1 (cut* or mutilat* or injur*)).tw.  67. ((stress adj1 (disorder* or behavio?r* or syndrom*)) or PTSD).tw.  68. ((substance or alcohol) adj1 (disorder* or abus* or dependenc* or addict*)).tw.  69. schizoid*.tw.  70. schizophren*.tw.  71. Schizotypal*.tw.  72. somatoform.tw.  73. suicid*.tw. |
| 74. **or/36-73** [MeSH and keywords for Mental Health] (830,099) |
| **Child, Adolescent and Young Adult MeSH and keywords:**  75. exp Infant/  76. exp child/  77. Adolescent/  78. exp Pediatrics/  79. Young Adult/  80. (infant* or neonate* or child* or adolescen* or teen* or youth or (young adj1 adult*) or p?ediatric* or juvenile*).tw. |
| 81. **or/73-78** [MeSH and keywords for pt <25yr] (3,108,037) |
| 82. **and/35,72,79** [Social media + Mental health + Youth] (3,143) |
| 83. remove duplicates from 82 (3,003)  84. limit 84 to yr="2013 -Current" (632) [UPDATE SEARCH ONLY] |
