# Supplementary material for: A Systematic Review of Social Media Use to Discuss and View Deliberate Self-Harm Acts
Source: PLoS One. 2016 May 18;11(5):e0155813. doi: 10.1371/journal.pone.0155813 (PMC4871432; doi:10.1371/journal.pone.0155813)
Supplement: S2 Appendix — (DOCX) [file pone.0155813.s002.docx]

**S2 Appendix. Mixed Methods Appraisal Tool Criteria^[[1]](#footnote-1)^**

1. Pluye P, Robert E, Cargo M, et al. Proposal: A mixed methods appraisal tool for systematic mixed studies reviews. 2011; <http://mixedmethodsappraisaltoolpublic.pbworks.com>. [↑](#footnote-ref-1)
