# Supplementary material for: A Systematic Review of Social Media Use to Discuss and View Deliberate Self-Harm Acts
Source: PLoS One. 2016 May 18;11(5):e0155813. doi: 10.1371/journal.pone.0155813 (PMC4871432; doi:10.1371/journal.pone.0155813)
Supplement: S3 Appendix — (DOCX) [file pone.0155813.s003.docx]

**S3 Appendix. List of Excluded Studies**

**Publication Type (n=108)**

1. LIFESKILLS training effective for adolescent drug prevention. Journal of Psychosocial Nursing & Mental Health Services. 2002;40(12):13.

2. Study shows benefits of Web-based campus drinking prevention. Alcoholism & Drug Abuse Weekly. 2003;15(17):5-6.

3. "Virtual therapist" to help depressed pupils. CPJ: Counselling & Psychotherapy Journal. 2004;15(7):22.

4. Support groups must pitch to young people. Australian Nursing Journal. 2005;13(4):37-.

5. Stealth Project Tracks the Effects of Pro-Anorexia Websites. Eating Disorders Review. 2005;16(3):5.

6. Eating disorders in young women: examining an online psychosocial intervention. Brown University Child & Adolescent Behavior Letter. 2006;22(10):1.

7. Reckitt Benckiser website seeks to prevent and treat prescription opioid abuse: buprenorphine meets the MySpace generation. Alcoholism & Drug Abuse Weekly. 2007;19(32):1-3.

8. Reach out central. The first mental health online game. Synergy (14427818). 2007(3):19.

9. Computer program reduces substance use in teen girls. Alcoholism & Drug Abuse Weekly. 2009;21(33):7.

10. Adolescent MH curriculum added to LearntobeHealthy.org. Mental Health Weekly. 2009;19(41):8.

11. Are Japan's hikikomori and depression in young people spreading abroad? Lancet. 2011;378(9796):1070.

12. Study provides support for sustained effects of computer-based prevention program. DATA: The Brown University Digest of Addiction Theory & Application. 2011;30(4):3-4.

13. Effectiveness of an online group course for adolescents and young adults with depressive symptoms: study protocol for a randomized controlled trial. Trials. 2011;12(1):196-201.

14. ...Facebook postings show beliefs/stereotypes about ADHD. Brown University Child & Adolescent Behavior Letter. 2011;27(8):2.

15. ...AAP clinical report on social media on youth. Brown University Child & Adolescent Behavior Letter. 2011;27(6):2.

16. Cool teens: a computerized treatment program for adolescents with anxiety disorders. Clinician's Research Digest. 2012;30(6):9.

17. Impact of media on children. Australian Nursing Journal. 2012;19(10):42-3.

18. Playing computer game reduced depression. Mental Health Practice. 2012;15(8):5.

19. Keep your eye on... online friendship and Internet addiction among teens. Brown University Child & Adolescent Behavior Letter. 2012;28(4):3.

20. Allison S, von Wahlde L, Shockley T, Gabbard GO. The development of the self in the era of the internet and role-playing fantasy games. American Journal of Psychiatry. 2006;163(3):381-5.

21. Alvarez A. IH8U: confronting cyberbullying and exploring the use of cybertools in teen dating relationships. Journal of Clinical Psychology. 2012;68(11):1205-15.

22. Andrew S, Cleary M, Jackson D. Facing a new frontier: safety in cyberspace and challenges for nursing. Journal of Psychosocial Nursing & Mental Health Services. 2012;50(8):4-5.

23. Barak A, Grohol JM. Current and Future Trends in Internet-Supported Mental Health Interventions. 2011. p. 155-96.

24. Becker K, Mayer M, Nagenborg M, El-Faddagh M, Schmidt MH. Parasuicide online: Can suicide websites trigger suicidal behaviour in predisposed adolescents? Nordic Journal of Psychiatry. 2004;58(2):111-4.

25. Bergstrom J, Andersson G, Lindefors J. [Scientific evidence for CBT-based self help in depression. Via Internet can more receive treatment]. Lakartidningen. 2009;106(5):282-6.

26. Birbal R, Maharajh H, Birbal R, Clapperton M, Jarvis J, Ragoonath A, et al. Cybersuicide and the adolescent population: challenges of the future? International Journal of Adolescent Medicine & Health. 2009;21(2):151-9.

27. Botella C, Mira A, Garcia-Palacios A, Quero S, Navarro M, Riera Lopez Del Amo A, et al. Smiling is fun: a Coping with Stress and Emotion Regulation Program. Studies in Health Technology & Informatics. 2012;181:123-7.

28. Braillon A, Dubois G. Web-based intervention and alcohol: who is upside down?... Kypri K, Saunders JB, Gallagher SJ. (2003) Acceptability of various brief intervention approaches for hazardous drinking among university students. Alcohol Alcohol. 38:626-8. Alcohol & Alcoholism. 2010;45(1):103.

29. Bross DC. Minimizing risks to children when they access the world wide web. Child Abuse & Neglect. 2005;29(7):749-52.

30. Brown JD, Keller SN. Can the mass media be healthy sex educators? Family Planning Perspectives. 2000;32(5):255-6.

31. Bulik CM, Marcus MD, Zerwas S, Levine MD, La Via M. The changing "weightscape" of bulimia nervosa. The American journal of psychiatry. 2012;169(10):1031-6.

32. Calear AL, Christensen H, Griffiths KM. Internet-based anxiety and depression prevention programs for children and adolescents. Bennett Levy, James [Ed]. 2010:393-8.

33. Cartwright GF, Finkelstein AB, Maennling MK. Caught in the web: Internet risks for children. Shore, Bruce M [Ed]. 2008;2:119-27.

34. Casacchia M, Pollice R, Roncone R. The narrative epidemiology of L'Aquila 2009 earthquake. Epidemiology & Psychiatric Science. 2012;21(1):13-21.

35. Cloud J. Bullied to death? Time. 2010;176(16):60-3.

36. Connors K, Connors K, Bernstein H. Innovative online smoking prevention education for pediatric providers, 'tween' girls, and their families. Journal of Communication in Healthcare. 2010;3(1):9-16.

37. Creighton C. Using social media to increase awareness of inhalant abuse. Journal of Communication in Healthcare. 2010;3(3/4):197-213.

38. de Wit M, Snoek F. The DAWN MIND Youth program. Pediatric Diabetes. 2009;10 Suppl 13:46-9.

39. Ehrlich S, Stegemann T. [Young Investigators in Biological child and adolescent psychiatry (YIBcap)--insights after one year of networking]. Zeitschrift fur Kinder-und Jugendpsychiatrie und Psychotherapie. 2007;35(1):59-63.

40. Elgan T, Hansson H, Zetterlind U, Kartengren N, Leifman H. Design of a Web-based individual coping and alcohol-intervention program (web-ICAIP) for children of parents with alcohol problems: study protocol for a randomized controlled trial. BMC Public Health. 2012;12:35.

41. Epstein JN. How can the internet help improve community-based pediatric ADHD care? Expert Review of Neurotherapeutics. 2012;12(5):501-3.

42. Forsthoff A, Hummel B, Moller H, Grunze H. [Suicidality and the Internet. Danger from new media]. Nervenarzt. 2006;77(3):343-5.

43. Gabarron E, Serrano JA, Wynn R, Armayones M. Avatars using computer/smartphone mediated communication and social networking in prevention of sexually transmitted diseases among North-Norwegian youngsters. BMC Medical Informatics & Decision Making. 2012;12(1):120-4.

44. Genuis SJG, Shelagh K. Internet interactions: adolescent health and cyberspace. Canadian Family Physician. 2005;51:329-31, 34-6.

45. Gleeson JF, Alvarez-Jimenez M, Lederman R. Moderated online social therapy for recovery from early psychosis. Psychiatric Services. 2012;63(7):719.

46. Goldschmidt KH, Catherine M. Can Technology Assist Teen Smokers to “Kick the Habit”? Journal of Pediatric Nursing. 2012;27(3):277-9.

47. Gorrindo T, Groves JE. Computer simulation and virtual reality in the diagnosis and treatment of psychiatric disorders. Academic Psychiatry. 2009;33(5):413-7.

48. Gound P, Grigaitis M, Thomas T. A computerized learning tool. American Journal of Nursing. 1998;98(11):56-8.

49. Gray NJ. Health information on the internet--a double-edged sword? Journal of Adolescent Health. 2008;42(5):432-3.

50. Griffiths K, Crisp D, Christensen H, Mackinnon A, Bennett K. The ANU WellBeing study: a protocol for a quasi-factorial randomised controlled trial of the effectiveness of an Internet support group and an automated Internet intervention for depression. BMC Psychiatry. 2010;10:20.

51. Hagihara A, Miyazaki S, Tarumi K. Internet use and suicide among younger age groups between 1989 and 2008 in Japan. Acta Psychiatrica Scandinavica. 2010;121(6):485; author reply -6.

52. Hill N. Adolescent substance use prevention interventions outside of classroom settings. Child & Adolescent Social Work Journal. 2008;25(6):451-67.

53. Hitosugi M, Nagai T, Tokudome S. A voluntary effort to save the youth suicide via the Internet in Japan. International Journal of Nursing Studies. 2007;44(1):157.

54. Hlaing WM. Sexual solicitation of youth on the Internet. JAMA. 2001;286(10):1176-7.

55. Hoek W, Aarts F, Schuurmans J, Cuijpers P. Who are we missing? Non-participation in an internet intervention trial for depression and anxiety in adolescents. European Child & Adolescent Psychiatry. 2012;21(10):593-5.

56. Hoffman HG. Virtual-reality therapy. Scientific American. 2004;291(2):58-65.

57. Iftene F. Internet use in adolescents: hobby or avoidance. Canadian Journal of Psychiatry - Revue Canadienne de Psychiatrie. 2004;49(11):789-90.

58. Kahn R, Arbib M. A Cybernetic Approach to Childhood Psychosis. Journal of Autism and Childhood Schizophrenia. 1973;3(3):261-73.

59. Killackey E, Anda A, Gibbs M, Alvarez-Jimenez M, Thompson A, Sun P, et al. Using internet enabled mobile devices and social networking technologies to promote exercise as an intervention for young first episode psychosis patients. BMC Psychiatry. 2011;11:80.

60. Kmietowicz Z. Website of patients' experiences launches new module on depression. BMJ. 2004;329(7471):878.

61. Kraft E. Cyberbullying: a worldwide trend of misusing technology to harass others. In: Morgan KB, C. A.;Spector, J. M., editor. Internet Society II: Advances in Education, Commerce & Governance. Wit Transactions on Information and Communication Technologies. 362006. p. 155-66.

62. Kutcher S. A web based education program in adolescent depression and suicide for primary care physicians European Psychiatry. 2009;24.

63. Le Heuzey M. [Social media, children and pediatricians]. Archives de Pediatrie. 2012;19(1):92-5.

64. Lekic K, J. K, Tratnjek P, Jereb B. Slovenian practice story: 10 years of e-counselling service for teenagers. Studies in Health Technology & Informatics. 2011;165:105-10.

65. Lindenberg CS. TeenSmart-Informa-T/Ayuda-T: an interactive Web-based bilingual intervention: overview... 35th Annual Communicating Nursing Research Conference/16th Annual WIN Assembly, "Health Disparities: Meeting the Challenge," held April 18-20, 2002, Palm Springs, California. Communicating Nursing Research. 2002;35:190.

66. Matano RA, Futa K, Wanat SF, Mussman LM, Leung CW. The Employee Stress and Alcohol Project: the development of a computer-based alcohol abuse prevention program for employees. Journal of Behavioral Health Services & Research. 2000;27(2):152-65.

67. McDermott E, Roen K, Piela A. Hard-to-reach youth online: Methodological advances in self-harm research. Sexuality Research & Social Policy: A Journal of the NSRC Jan. 2013(Pagination):No Pagination Specified.

68. Mehlum L. The internet, suicide, and suicide prevention. Crisis: Journal of Crisis Intervention & Suicide. 2000;21(4):186-8.

69. Merrick-Kenig E, Merrick J. Cybersuicide. International Journal of Adolescent Medicine & Health. 2009;21(2):135-6.

70. Messina E, Iwasaki Y. Internet use and self-injurious behaviors among adolescents and young adults: an interdisciplinary literature review and implications for health professionals. Cyberpsychology, behavior and social networking. 2011;14(3):161-8.

71. Miller J. Finding Support Online: Parents are Finding Comfort and Support in Virtual Hugs. Exceptional Parent. 2006;36(10):42-4.

72. Muller A. Virtual communities and translation into physical reality in the 'It Gets Better' project. Journal of Media Practice. 2011;12(3):269-77.

73. Murray A. Talking 2 ourselves. Mental Health Today. 2005:12-3.

74. National Center for ME, Children. Helping students deal with cyberbullying. NASN school nurse. 2009;24(5):200-3.

75. O'Keeffe G, Clarke-Pearson K, Communications MCo. The impact of social media on children, adolescents, and families. Pediatrics. 2011;127(4):800-4.

76. Ogburn K, Messias E, Buckley P. New-age patient communications through social networks. General Hospital Psychiatry. 2011;33(2):200.e1-3.

77. Ozawa-De Silva C. Shared death: self, sociality and internet group suicide in Japan. Transcultural Psychiatry. 2010;47(3):392-418.

78. Patton G, Sawyer S. Media and young minds. Medical Journal of Australia. 2000;173(11-12):570-1.

79. Pector EA. Sharing Losses Online: Do Internet Support Groups Benefit the Bereaved? International Journal of Childbirth Education. 2012;27(2):19-25.

80. Penn DL, Simpson LE, Leggett S, Edie G, Wood L. The development of a Web site to promote the mental and physical health of sons and daughters of Vietnam veterans of Australia. Journal of Consumer Health on the Internet. 2006;10(4):45-63.

81. Plusquellec M. [Are virtual worlds a threat to the mental health of children and adolescents?]. Archives de Pediatrie. 2000;7(2):209-10.

82. Potera C. YouTube self-harm videos under scrutiny. American Journal of Nursing. 2011;111(6):20.

83. Pujazon-Zazik M, Park M. To tweet, or not to tweet: gender differences and potential positive and negative health outcomes of adolescents' social internet use. American Journal of Mens Health. 2010;4(1):77-85.

84. Rabin RC. Behavior: Videos of Self-Injury Find an Audience. New York Times. 2011:6.

85. Rapple B. [When patients use internet education]. Krankenpflege - Soins Infirmiers. 2011;104(7):20-3.

86. Rice E, Karnik NS. Network science and social media. Journal of the American Academy of Child & Adolescent Psychiatry. 2012;51(6):563-5.

87. Rickwood DJ. Promoting youth mental health through computer-mediated communication. International Journal of Mental Health Promotion. 2010;12(3):32-44.

88. Riegel DL. Boyhood sexual experiences with older males: using the Internet for behavioral research. Archives of Sexual Behavior. 2009;38(5):626-30.

89. Rizzo A, Parsons T, Lange B, Kenny P, Buckwalter J, Rothbaum B, et al. Virtual reality goes to war: a brief review of the future of military behavioral healthcare. Journal of Clinical Psychology in Medical Settings. 2011;18(2):176-87.

90. Sagedal LR, Øverby NC, Lohne-Seiler H, Bere E, Torstveit MK, Henriksen T, et al. Study protocol: fit for delivery - can a lifestyle intervention in pregnancy result in measurable health benefits for mothers and newborns? A randomized controlled trial. BMC Public Health. 2013;13(1):1-9.

91. Scharer K. Internet social support for parents: the state of science. Journal of Child & Adolescent Psychiatric Nursing. 2005;18(1):26-35.

92. Smith DJ. S29-03 - Web-based psychoeducation for bipolar disorder. European Psychiatry. 2010;25:20.

93. Spriggs M. Consent in cyberspace: Internet-based research involving young people. Monash Bioethics Review. 2009;28(4):32.1-15.

94. Stochel M, Janas-Kozik M. [Friends of virtual Ana--the phenomenon of pro-anorexia in the Internet]. Psychiatria Polska. 2010;44(5):693-702.

95. Summerskill B. Online social networks and wellbeing. Lancet. 2009;374(9689):514.

96. Suzuki K, Asaga R, Sourander A, Hoven C, Mandell DJ. Cyberbullying and adolescent mental health. International Journal of Adolescent Medicine & Health. 2012;24(1):27-35.

97. Taylor S. Adolescent angst, internet innovations and the adult children of divorce. Perspectives in Public Health. 2010;130(2):50.

98. Thongpriwan V, McElmurry BJ. Comparisons between Thai adolescent voices and Thai adolescent health literature. Journal of School Health. 2006;76(2):47-51.

99. Trudeau KJ, Quinonez L. Technology. Online drug education attracts young teens. Addiction Professional. 2008;6(4):42-3.

100. Vandermersch N, Venisse J, Chassevent A. [Kimberly and her blogs]. Archives de Pediatrie. 2008;15(5):806-7.

101. Vigerland S, Thulin U, Ljotsson B, Svirsky L, Bengtsson O, Lindefors N, et al. [Computerized therapy for anxiety disorders should also be considered for children and adolescents. Can give better access to evidence-based treatment]. Lakartidningen. 2013;110(3):92-4.

102. Wallace P, Linke S, Murray E, McCambridge J, Thompson S. A randomized controlled trial of an interactive Web-based intervention for reducing alcohol consumption. Journal of Telemedicine & Telecare. 2006;12 Suppl 1:52-4.

103. Webb M, Burns J, Collin P. Providing online support for young people with mental health difficulties: challenges and opportunities explored. Early intervention in psychiatry. 2008;2(2):108-13.

104. Webster ST. Facebook and eating disorders in adolescent girls. IDEA Fitness Journal. 2011;8(5):59.

105. Williams III DS. The PatientsLikeMe® Multiple Sclerosis Community: Using online marketing to shift the health data privacy paradigm. Journal of Communication in Healthcare. 2010;3(1):48-61.

106. Williams SG, Godfrey AJ. What is Cyberbullying & How Can Psychiatric-Mental Health Nurses Recognize It? Journal of Psychosocial Nursing & Mental Health Services. 2011;49(10):36-41.

107. Woolderink M, Smit F, van der Zanden R, Beecham J, Knapp M, Paulus A, et al. Design of an internet-based health economic evaluation of a preventive group-intervention for children of parents with mental illness or substance use disorders. BMC Public Health. 2010;10:470.

108. Zabinski MF. The Internet-based My Body, My Life: Body Image Program for adolescent girls improves body image and disordered eating. Evidence Based Mental Health. 2008;11(1):23.

**Intervention (n=381)**

1. Aardema F, O'Connor K, Cote S, Taillon A. Virtual reality induces dissociation and lowers sense of presence in objective reality. Cyberpsychology, behavior and social networking. 2010;13(4):429-35.

2. Abbott J-AM, Kaldo V, Klein B, Austin D, Hamilton C, Piterman L, et al. A cluster randomised trial of an internet-based intervention program for tinnitus distress in an industrial setting. Cognitive Behaviour Therapy. 2009;38(3):162-73.

3. Adams J, Rodham K, Gavin J. Investigating the "self" in deliberate self-harm. Qualitative Health Research. 2005;15(10):1293-309.

4. Adams R, Finn P, Moes E, Flannery K, Rizzo AS. Distractibility in Attention/Deficit/ Hyperactivity Disorder (ADHD): the virtual reality classroom. Child Neuropsychology. 2009;15(2):120-35.

5. An LC, Schillo BA, Saul JE, Wendling AH, Klatt CM, Berg CJ, et al. Utilization of smoking cessation informational, interactive, and online community resources as predictors of abstinence: cohort study. Journal of Medical Internet Research. 2008;10(5):e55-e.

6. Andén-Papadopoulos K. US Soldiers Imaging the Iraq War on YouTube. Popular Communication. 2009;7(1):17-27.

7. Andersson G, Waara J, Jonsson U, Malmaeus F, Carlbring P, Ost L-G. Internet-based self-help versus one-session exposure in the treatment of spider phobia: a randomized controlled trial. Cognitive Behaviour Therapy. 2009;38(2):114-20.

8. Andrewes DG, O'Connor P, Mulder C, McLennan J, Derham H, Weigall S, et al. Computerised psychoeducation for patients with eating disorders. Australian & New Zealand Journal of Psychiatry. 1996;30(4):492-7.

9. Arpin-Cribbie C, Irvine J, Ritvo P. Web-based cognitive-behavioral therapy for perfectionism: a randomized controlled trial. Psychotherapy Research. 2012;22(2):194-207.

10. Bae J, Wolpin S, Kim E, Lee S, Yoon S, An K. Development of a user-centered health information service system for depressive symptom management. Nursing & Health Sciences. 2009;11(2):185-93.

11. Baer S, Saran K, Green DA, Hong I. Electronic media use and addiction among youth in psychiatric clinic versus school populations. Canadian Journal of Psychiatry - Revue Canadienne de Psychiatrie. 2012;57(12):728-35.

12. Balmford J, Borland R, Li L, Ferretter I. Usage of an Internet smoking cessation resource: the Australian QuitCoach. Drug & Alcohol Review. 2009;28(1):66-72.

13. Basch E, Artz D, Dulko D, Scher K, Sabbatini P, Hensley M, et al. Patient online self-reporting of toxicity symptoms during chemotherapy. Journal of Clinical Oncology. 2005;23(15):3552-61.

14. Baumgartner SE, Sumter SR, Peter J, Valkenburg PM. Identifying teens at risk: developmental pathways of online and offline sexual risk behavior. Pediatrics. 2012;130(6):e1489-96.

15. Beamish N, Cannan P, Fujiyama H, Matthews A, Spiranovic C, Briggs K, et al. Evaluation of an Online Youth Ambassador Program to Promote Mental Health. Youth Studies Australia. 2011;30(2):41-7.

16. Beck CT. Benefits of participating in Internet interviews: Women helping women. Qualitative Health Research. 2005;15(3):411-22.

17. Beckman L, Hagquist C, Hellström L. Does the association with psychosomatic health problems differ between cyberbullying and traditional bullying? Emotional & Behavioural Difficulties. 2012;17(3/4):421-34.

18. Belfort EL, Mezzacappa E, Ginnis K. Similarities and differences among adolescents who communicate suicidality to others via electronic versus other means: A pilot study. Adolescent Psychiatry. 2012;2(3):258-62.

19. Bell MD, Weinstein A. Simulated job interview skill training for people with psychiatric disability: feasibility and tolerability of virtual reality training. Schizophrenia Bulletin. 2011;37 Suppl 2:S91-7.

20. Bell RA, Paterniti DA, Azari R, Duberstein PR, Epstein RM, Rochlen AB, et al. Encouraging patients with depressive symptoms to seek care: a mixed methods approach to message development. Patient Education & Counseling. 2010;78(2):198-205.

21. Bell RA, Taylor LD, Kravitz RL. Do antidepressant advertisements educate consumers and promote communication between patients with depression and their physicians? Patient Education & Counseling. 2010;81(2):245-50.

22. Berg CJ, Schauer GL. Results of a feasibility and acceptability trial of an online smoking cessation program targeting young adult nondaily smokers. Journal Of Environmental & Public Health. 2012;2012:248541-.

23. Bertocci MA. Media use by children and adolescents with and without depression: Going it alone? Dissertation Abstracts International Section A: Humanities and Social Sciences. 2011;72(5-A):1535.

24. Bessell A, Clarke A, Harcourt D, Moss TP, Rumsey N. Incorporating user perspectives in the design of an online intervention tool for people with visible differences: face IT. Behavioural & Cognitive Psychotherapy. 2010;38(5):577-96.

25. Bessell A, Brough V, Clarke A, Harcourt D, Moss TP, Rumsey N. Evaluation of the effectiveness of Face IT, a computer-based psychosocial intervention for disfigurement-related distress. Psychology, Health & Medicine. 2012;17(5):565-77.

26. Billings DW, Cook RF, Hendrickson A, Dove DC. A web-based approach to managing stress and mood disorders in the workforce. Journal of Occupational & Environmental Medicine. 2008;50(8):960-8.

27. Bingham CR, Barretto AI, Walton MA, Bryant CM, Shope JT, Raghunathan TE. Efficacy of a web-based, tailored, alcohol prevention/intervention program for college students: initial findings. Journal of American College Health. 2010;58(4):349-56.

28. Bishop D, Bryant KS, Giles SM, Hansen WB, Dusenbury L. Simplifying the delivery of a prevention program with web-based enhancements. Journal of Primary Prevention. 2006;27(4):433-44.

29. Bobkowski PS, Brown JD, Neffa DR. "Hit me up and we can get down": US youths' risk behaviors and sexual self-disclosure in MySpace profiles. Journal of Children and Media. 2012;6(1):119-34.

30. Bolding G, Davis M, Hart G, Sherr L, Elford J. Gay men who look for sex on the Internet: is there more HIV/STI risk with online partners? AIDS. 2005;19(9):961-8.

31. Borzekowski DLG, Leith J, Medoff DR, Potts W, Dixon LB, Balis T, et al. Use of the Internet and Other Media for Health Information Among Clinic Outpatients With Serious Mental Illness. Psychiatric Services. 2009;60(9):1265-8.

32. Bosworth K, Espelage D, DuBay T. A computer-based violence prevention Intervention for young adolescents: Pilot study. Adolescence. 1998;33(132):785.

33. Botella C, Garcia-Palacios A, Guillen V, Banos RM, Quero S, Alcaniz M. An adaptive display for the treatment of diverse trauma PTSD victims. Cyberpsychology, behavior and social networking. 2010;13(1):67-71.

34. Bowen AM, Williams ML, Daniel CM, Clayton S. Internet based HIV prevention research targeting rural MSM: feasibility, acceptability, and preliminary efficacy. Journal of Behavioral Medicine. 2008;31(6):463-77.

35. Bowen DJ, Henderson PN, Harvill J, Buchwald D. Short-term Effects of a Smoking Prevention Website in American Indian Youth. Journal of Medical Internet Research. 2012;14(3):e81-e.

36. Bragadottir H. Developing a computer-mediated support group intervention for parents whose children have been diagnosed with cancer: University of Iowa; 2004.

37. Braithwaite SR, Fincham FD. ePREP: Computer based prevention of relationship dysfunction, depression and anxiety. Journal of Social and Clinical Psychology. 2007;26(5):609-22.

38. Brendryen H, Drozd F, Kraft P. A digital smoking cessation program delivered through internet and cell phone without nicotine replacement (happy ending): randomized controlled trial. Journal of Medical Internet Research. 2008;10(5):e51.

39. Budney AJ, Fearer S, Walker DD, Stanger C, Thostenson J, Grabinski M, et al. An initial trial of a computerized behavioral intervention for cannabis use disorder. Drug & Alcohol Dependence. 2011;115(1-2):74-9.

40. Buffardi LE, Campbell WK. Narcissism and social networking Web sites. Personality & Social Psychology Bulletin. 2008;34(10):1303-14.

41. Buller DB, Borland R, Woodall WG, Hall JR, Hines JM, Burris-Woodall P, et al. Randomized trials on Consider This, a tailored, Internet-delivered smoking prevention program for adolescents. Health Education & Behavior. 2008;35(2):260-81.

42. Buller DB, Woodall WG, Zimmerman DE, Slater MD, Heimendinger J, Waters E, et al. Randomized Trial on the 5 a Day, the Rio Grande Way Website, A Web-based Program to Improve Fruit and Vegetable Consumption in Rural Communities. Journal of Health Communication. 2008;13(3):230-49.

43. Burgess Dowdell E. Risky internet behaviors of middle-school students: communication with online strangers and offline contact. CIN: Computers, Informatics, Nursing. 2011;29(6):352-9.

44. Calam R, Sanders MR, Miller C, Sadhnani V, Carmont S. Can technology and the media help reduce dysfunctional parenting and increase engagement with preventative parenting interventions? Child Maltreatment. 2008;13(4):347-61.

45. Calear AL, Christensen H, Mackinnon A, Griffiths KM, O'Kearney R. The YouthMood Project: A Cluster Randomized Controlled Trial of an Online Cognitive Behavioral Program with Adolescents. Journal of consulting and clinical psychology. 1021;77(6):1021-32.

46. Callahan A, Inckle K. Cybertherapy or psychobabble? A mixed methods study of online emotional support. British Journal of Guidance & Counselling. 2012;40(3):261-78.

47. Campbell AJ, Cumming SR, Hughes I. Internet use by the socially fearful: addiction or therapy? Cyberpsychology & Behavior. 2006;9(1):69-81.

48. Campbell M, Spears B, Slee P, Butler D, Kift S. Victims' Perceptions of Traditional and Cyberbullying, and the Psychosocial Correlates of Their Victimisation. Emotional and Behavioural Difficulties. 2012;17(3):389-401.

49. Cardi V, Krug I, Perpina C, Mataix-Cols D, Roncero M, Treasure J. The use of a nonimmersive virtual reality programme in anorexia nervosa: a single case-report. European Eating Disorders Review. 2012;20(3):240-5.

50. Carpenter EM, Frankel F, Marina M, Duan N, Smalley SL. Internet Treatment Delivery of Parent-Adolescent Conflict Training for Families with an ADHD Teen: A Feasibility Study. Child & Family Behavior Therapy. 2004;26(3):1-20.

51. Carrard I, Fernandez-Aranda F, Lam T, Nevonen L, Liwowsky I, Volkart AC, et al. Evaluation of a guided internet self-treatment programme for bulimia nervosa in several European countries. European Eating Disorders Review. 2011;19(2):138-49.

52. Cetin B, Eroglu Y, Peker A, Akbaba S, Pepsoy S. The Investigation of Relationship among Relational-Interdependent Self-Construal, Cyberbullying, and Psychological Disharmony in Adolescents: An Investigation of Structural Equation Modelling. Educational Sciences: Theory and Practice. 2012;12(2):646-53.

53. Chiasson MA, Hirshfield S, Remien RH, Humberstone M, Wong T, Wolitski RJ. A comparison of on-line and off-line sexual risk in men who have sex with men: an event-based on-line survey. Journal of Acquired Immune Deficiency Syndromes: JAIDS. 2007;44(2):235-43.

54. Chiauzzi E, Brevard J, Thum C, Decembrele S, Lord S. MyStudentBody-Stress: an online stress management intervention for college students.[Erratum appears in J Health Commun. 2008 Dec;13(8):827 Note: Thurn, Christina [corrected to Thum, Christina]]. Journal of Health Communication. 2008;13(6):555-72.

55. Christensen H, Griffiths KM, Jorm AF. Delivering interventions for depression by using the internet: randomised controlled trial. BMJ. 2004;328(7434):265.

56. Christensen H, Leach LS, Barney L, Mackinnon AJ, Griffiths KM. The effect of web based depression interventions on self reported help seeking: randomised controlled trial [ISRCTN77824516]. BMC Psychiatry. 2006;6:13.

57. Chu JL, Snider CE. Use of a social networking web site for recruiting canadian youth for medical research. Journal of Adolescent Health Jan. 2013(Pagination):No Pagination Specified.

58. Collard DC, Chinapaw MJ, Verhagen EA, Bakker I, van Mechelen W. Effectiveness of a school-based physical activity-related injury prevention program on risk behavior and neuromotor fitness a cluster randomized controlled trial. The International Journal of Behavioral Nutrition and Physical Activity. 2010;7:9.

59. Court J, Carr-Gregg M, Bergh C, Brodin U, Callmar M, Ejderhamn J, et al. An innovative treatment programme for anorexia nervosa. Journal of Paediatrics & Child Health. 2005;41(5-6):305-6.

60. Crits-Christoph P, Ring-Kurtz S, McClure B, Temes C, Kulaga A, Gallop R, et al. A randomized controlled study of a web-based performance improvement system for substance abuse treatment providers. Journal of Substance Abuse Treatment. 2010;38(3):251-62.

61. Croom K, Lewis D, Marchell T, Lesser ML, Reyna VF, Kubicki-Bedford L, et al. Impact of an online alcohol education course on behavior and harm for incoming first-year college students: short-term evaluation of a randomized trial. Journal of American College Health. 2009;57(4):445-54.

62. Crutzen R, de Nooijer J, Candel M, de Vries NK. Adolescents who intend to change multiple health behaviours choose greater exposure to an internet-delivered intervention. Journal of Health Psychology. 2008;13(7):906-11.

63. da Silva LAV, Iriart JAB. The meanings and practices of barebacking among Brazilian internet users. Sociology of Health & Illness. 2012;34(5):651-64.

64. Daley ML, Becker DF, Flaherty LT, Harper G, King RA, Lester P, et al. Case study: the internet as a developmental tool in an adolescent boy with psychosis. Journal of the American Academy of Child & Adolescent Psychiatry. 2005;44(2):187-90.

65. Darcy AM, Dooley B. A clinical profile of participants in an online support group. European Eating Disorders Review. 2007;15(3):185-95.

66. David C, Cappella JN, Fishbein M. The social diffusion of influence among adolescents: Group interaction in a chat room environment about antidrug advertisements. Communication Theory. 2006;16(1):118-40.

67. Davis WM, Shoveller JA, Oliffe JL, Gilbert M. Young people's perspectives on the use of reverse discourse in web-based sexual-health interventions. Culture, Health & Sexuality. 2012;14(9):1065-79.

68. de Graaf LE, Gerhards SAH, Arntz A, Riper H, Metsemakers JFM, Evers SMAA, et al. Clinical effectiveness of online computerised cognitive-behavioural therapy without support for depression in primary care: randomised trial. British Journal of Psychiatry. 2009;195(1):73-80.

69. de Graaf LE, Huibers MJH, Riper H, Gerhards SAH, Arntz A. Use and acceptability of unsupported online computerized cognitive behavioral therapy for depression and associations with clinical outcome. Journal of Affective Disorders. 2009;116(3):227-31.

70. de Graaf LE, Hollon SD, Huibers MJH. Predicting outcome in computerized cognitive behavioral therapy for depression in primary care: A randomized trial. Journal of Consulting & Clinical Psychology. 2010;78(2):184-9.

71. Deitz DK, Cook RF, Billings DW, Hendrickson A. A web-based mental health program: reaching parents at work. Journal of Pediatric Psychology. 2009;34(5):488-94.

72. Del Rey R, Casas JA, Ortega R. El programa ConRed, una práctica basada en la evidencia. (Spanish). The ConRed Program, an Evidence-based Practice (English). 2012;20(39):129-38.

73. Di Noia J, Schwinn TM, Dastur ZA, Schinke SP. The relative efficacy of pamphlets, CD-ROM, and the Internet for disseminating adolescent drug abuse prevention programs: an exploratory study. Preventive Medicine. 2003;37(6 Pt 1):646-53.

74. Didden R, Scholte RHJ, Korzilius H, de Moor JM, Vermeulen A, O'Reilly M, et al. Cyberbullying among students with intellectual and developmental disability in special education settings. Developmental Neurorehabilitation. 2009;12(3):146-51.

75. Donelle L, Hoffman-Goetz L. An Exploratory Study of Canadian Aboriginal Online Health Care Forums. Health Communication. 2008;23(3):270-81.

76. Donovan E, Wood M, Frayjo K, Black RA, Surette DA. A randomized, controlled trial to test the efficacy of an online, parent-based intervention for reducing the risks associated with college-student alcohol use. Addictive Behaviors. 2012;37(1):25-35.

77. Dooks P, McQuestion M, Goldstein D, Molassiotis A. Experiences of patients with laryngectomies as they reintegrate into their community. Supportive Care in Cancer. 2012;20(3):489-98.

78. Doran M, Smith A, Hoppel A, Morse L, Edwards M, Hamilton J, et al. Informing Students about the Variable Quality of Psychological Internet Resources: Sites Targeting Substance Abuse Problems, Emotional Disorders, and the Needs of Parents. 1998.

79. Dow MG, Kenardy JA, Johnston DW, Newman MG, Taylor CB, Thomson A. Prognostic indices with brief and standard CBT for panic disorder: I. Predictors of outcome. Psychological Medicine. 2007;37(10):1493-502.

80. Dowdell EB. Risky Internet Behaviors of Middle-School Students: Communication With Online Strangers and Offline Contact. CIN: Computers, Informatics, Nursing. 2013;31:TC26-33.

81. Dressing H, Anders A, Gallas C, Bailer J. [Cyberstalking: prevalence and impact on victims]. Psychiatrische Praxis. 2011;38(7):336-41.

82. Drost LM, Cuijpers P, Schippers GM. Developing an interactive website for adolescents with a mentally ill family member. Clinical Child Psychology & Psychiatry. 2011;16(3):351-64.

83. Drost LM, Sytema S, Schippers GM. Internet support for adolescents with a mentally ill family member. Psychiatric Services. 2011;62(3):322.

84. Duquette CA, Stodel EJ, Fullarton S, Hagglund K. Educational advocacy among adoptive parents of adolescents with fetal alcohol spectrum disorder. International Journal of Inclusive Education. 2012;16(11):1203-21.

85. Eagen T. Parent and adolescent Internet use, perception, and regulation: A dyadic analysis. Dissertation Abstracts International Section A: Humanities and Social Sciences. 2008;69(6-A):2233.

86. Egan KG, Moreno MA. Alcohol references on undergraduate males' Facebook profiles. American Journal of Mens Health. 2011;5(5):413-20.

87. Elf M, Skarsater I, Krevers B. 'The web is not enough, it's a base'--an explorative study of what needs a web-based support system for young carers must meet. Informatics for health & social care. 2011;36(4):206-19.

88. Enebrink P, Hogstrom J, Forster M, Ghaderi A. Internet-based parent management training: a randomized controlled study. Behaviour Research & Therapy. 2012;50(4):240-9.

89. Fang L, Schinke SP, Cole KCA. Preventing substance use among early Asian-American adolescent girls: initial evaluation of a web-based, mother-daughter program. Journal of Adolescent Health. 2010;47(5):529-32.

90. Fang L, Schinke SP. Two-Year Outcomes of a Randomized, Family-Based Substance Use Prevention Trial for Asian American Adolescent Girls. Psychology of Addictive Behaviors Dec. 2012(Pagination):No Pagination Specified.

91. Ferguson CJ, Munoz ME, Garza A, Galindo M. Concurrent and prospective analyses of peer, television and social media influences on body dissatisfaction, eating disorder symptoms and life satisfaction in adolescent girls. Journal of Youth and Adolescence Jan. 2013(Pagination):No Pagination Specified.

92. Fernandez-Aranda F, Nunez A, Martinez C, Krug I, Cappozzo M, Carrard I, et al. Internet-based cognitive-behavioral therapy for bulimia nervosa: a controlled study. Cyberpsychology & Behavior. 2009;12(1):37-41.

93. Ferrer-Garcia M, Gutierrez-Maldonado J. Virtual reality exposure in patients with eating disorders: influence of symptom severity and presence. Studies in Health Technology & Informatics. 2011;167:80-5.

94. Fikar CR, Keith L. Information needs of gay, lesbian, bisexual, and transgendered health care professionals: results of an Internet survey. Journal of the Medical Library Association. 2004;92(1):56-65.

95. Fleming T, Dixon R, Frampton C, Merry S. A pragmatic randomized controlled trial of computerized CBT (SPARX) for symptoms of depression among adolescents excluded from mainstream education. Behavioural and Cognitive Psychotherapy. 2012;40(5):529-41.

96. Flessner CA, Mouton-Odum S, Stocker AJ, Keuthen NJ. StopPicking.com: Internet-based treatment for self-injurious skin picking. Dermatology Online Journal. 2007;13(4):3.

97. Fraser T, McRobbie H, Bullen C, Whittaker R, Barlow D. Acceptability and outcome of an Internet-based smoking cessation programme. International Journal of Tuberculosis & Lung Disease. 2010;14(1):113-8.

98. Friberg P, Hagquist C, Osika W. Self-perceived psychosomatic health in Swedish children, adolescents and young adults: an internet-based survey over time. Bmj Open. 2012;2(4).

99. Fridrici M, Lohaus A. Stress-Prevention in Secondary Schools: Online- versus Face-to-Face-Training. Health Education. 2009;109(4):299-313.

100. Fujioka N, Kobayashi T, Turale S. Short-term behavioral changes in pregnant women after a quit-smoking program via e-learning: A descriptive study from Japan. Nursing & Health Sciences. 2012;14(3):304-11.

101. Gackenbach J, Sample T, Mandel G, Tomashewsky M. Dream and Blog Content Analysis of a Long Term Diary of a Video Game Player With Obsessive Compulsive Disorder. Dreaming. 2011;21(2):124-47.

102. Garner M, Mogg K, Bradley BP. Fear-relevant selective associations and social anxiety: absence of a positive bias. Behaviour Research & Therapy. 2006;44(2):201-17.

103. Gass K, Hoff CC, Stephenson R, Sullivan PS. Sexual agreements in the partnerships of internet-using men who have sex with men. AIDS Care. 2012;24(10):1255-63.

104. Gerhards SAH, de Graaf LE, Jacobs LE, Severens JL, Huibers MJH, Arntz A, et al. Economic evaluation of online computerised cognitive-behavioural therapy without support for depression in primary care: randomised trial. British Journal of Psychiatry. 2010;196(4):310-8.

105. Gerrits RS, van der Zanden RA, Visscher RF, Conijn BP. Master your mood online: A preventive chat group intervention for adolescents. AeJAMH (Australian e-Journal for the Advancement of Mental Health). 2007;6(3):1-11.

106. Gilbert LK, Temby JRE, Rogers SE. Evaluating a teen STD prevention Web site. Journal of Adolescent Health. 2005;37(3):236-42.

107. Glider P, Midyett SJ, Mills-Novoa B, Johannessen K, Collins C. Challenging the collegiate rite of passage: a campus-wide social marketing media campaign to reduce binge drinking. Journal of Drug Education. 2001;31(2):207-20.

108. Goodman M, Patil U, Triebwasser J, Hoffman P, Weinstein ZA, New A. Parental burden associated with borderline personality disorder in female offspring. Journal of Personality Disorders. 2011;25(1):59-74.

109. Gowen K, Deschaine M, Gruttadara D, Markey D. Young adults with mental health conditions and social networking websites: seeking tools to build community. Psychiatric Rehabilitation Journal. 2012;35(3):245-50.

110. Grov C. Risky sex- and drug-seeking in a probability sample of men-for-men online bulletin board postings. AIDS & Behavior. 2010;14(6):1387-92.

111. Grunwald M, Wesemann D. Individual use of online-consulting for persons affected with eating disorders and their relatives -- evaluation of an online consulting service. European Eating Disorders Review. 2006;14(4):218-25.

112. Gulliver A, Griffiths KM, Christensen H, Mackinnon A, Calear AL, Parsons A, et al. Internet-based interventions to promote mental health help-seeking in elite athletes: an exploratory randomized controlled trial. Journal of Medical Internet Research. 2012;14(3):e69.

113. Gutierrez-Maldonado J, Ferrer-Garcia M, Caqueo-Urizar A, Letosa-Porta A. Assessment of emotional reactivity produced by exposure to virtual environments in patients with eating disorders. Cyberpsychology & Behavior. 2006;9(5):507-13.

114. Haber P, Iskander J, Walton K, Campbell SR, Kohl KS. Internet-based reporting to the vaccine adverse event reporting system: a more timely and complete way for providers to support vaccine safety. Pediatrics. 2011;127 Suppl 1:S39-44.

115. Hall MJ, Tidwell WC. Internet recovery for substance abuse and alcoholism: an exploratory study of service users. Journal of Substance Abuse Treatment. 2003;24(2):161-7.

116. Hall W, Irvine V. E-communication among mothers of infants and toddlers in a community-based cohort: a content analysis. Journal of Advanced Nursing. 2009;65(1):175-83.

117. Harper K, Sperry S, Thompson JK. Viewership of pro-eating disorder websites: association with body image and eating disturbances. International Journal of Eating Disorders. 2008;41(1):92-5.

118. Harris KM, McLean JP, Sheffield J. Examining suicide-risk individuals who go online for suicide-related purposes. Archives of Suicide Research. 2009;13(3):264-76.

119. Hawkins RP, et al. Reaching Hard-to-Reach Populations: Interactive Computer Programs as Public Information Campaigns for Adolescents. Journal of Communication. 1987;37(2):8-28.

120. Hay C, Meldrum R. Bullying victimization and adolescent self-harm: testing hypotheses from general strain theory. Journal of Youth & Adolescence. 2010;39(5):446-59.

121. Hedman E, Andersson E, Ljotsson B, Andersson G, Ruck C, Lindefors N. Cost-effectiveness of Internet-based cognitive behavior therapy vs. cognitive behavioral group therapy for social anxiety disorder: results from a randomized controlled trial. Behaviour Research & Therapy. 2011;49(11):729-36.

122. Hedman E, Andersson G, Ljotsson B, Andersson E, Ruck C, Mortberg E, et al. Internet-based cognitive behavior therapy vs. cognitive behavioral group therapy for social anxiety disorder: a randomized controlled non-inferiority trial. PLoS ONE [Electronic Resource]. 2011;6(3):e18001.

123. Hedman E, Andersson E, Ljotsson B, Andersson G, Schalling M, Lindefors N, et al. Clinical and genetic outcome determinants of Internet- and group-based cognitive behavior therapy for social anxiety disorder. Acta Psychiatrica Scandinavica. 2012;126(2):126-36.

124. Helweg-Larsen K, Schutt N, Larsen HB. Predictors and protective factors for adolescent Internet victimization: results from a 2008 nationwide Danish youth survey. Acta Paediatrica. 2012;101(5):533-9.

125. Hester RK, Delaney HD, Campbell W. The college drinker's check-up: outcomes of two randomized clinical trials of a computer-delivered intervention. Psychology of Addictive Behaviors. 2012;26(1):1-12.

126. Hidaka Y, Ichikawa S, Koyano J, Urao M, Yasuo T, Kimura H, et al. Substance use and sexual behaviours of Japanese men who have sex with men: a nationwide internet survey conducted in Japan. BMC Public Health. 2006;6:239.

127. Hidaka Y, Operario D. Attempted suicide, psychological health and exposure to harassment among Japanese homosexual, bisexual or other men questioning their sexual orientation recruited via the internet. Journal of Epidemiology & Community Health. 2006;60(11):962-7.

128. Hirai M, Skidmore ST, Clum GA, Dolma S. An investigation of the efficacy of online expressive writing for trauma-related psychological distress in Hispanic individuals. Behavior Therapy. 2012;43(4):812-24.

129. Hoek W, Marko M, Fogel J, Schuurmans J, Gladstone T, Bradford N, et al. Randomized controlled trial of primary care physician motivational interviewing versus brief advice to engage adolescents with an Internet-based depression prevention intervention: 6-month outcomes and predictors of improvement. Translational Research: The Journal Of Laboratory & Clinical Medicine. 2011;158(6):315-25.

130. Hollander EM. Cyber community in the valley of the shadow of death. Journal of Loss and Trauma. 2001;6(2):135-46.

131. Holtslander L, Kornder N, Letourneau N, Turner H, Paterson B. Finding straight answers: identifying the needs of parents and service providers of adolescents with type 1 diabetes to aid in the creation of an online support intervention. Journal of Clinical Nursing. 2012;21(17/18):2419-28.

132. Horgan A, Sweeney J. Young students' use of the Internet for mental health information and support. Journal of Psychiatric & Mental Health Nursing. 2010;17(2):117-23.

133. Horvath KJ, Bowen AM, Williams ML. Virtual and physical venues as contexts for HIV risk among rural men who have sex with men. Health Psychology. 2006;25(2):237-42.

134. Horvath KJ, Oakes JM, Rosser BRS. Sexual negotiation and HIV serodisclosure among men who have sex with men with their online and offline partners. Journal of Urban Health. 2008;85(5):744-58.

135. Huber JS. The mediating effect of sibling warmth on parental stress in families with children who have attention deficit hyperactivity disorder. Dissertation Abstracts International Section A: Humanities and Social Sciences. 2011;71(8-A):3053.

136. Hudson DB, Elek SM, Westfall JR, Grabau A, Fleck MO. Young Parents Project: A 21st-Century Nursing Intervention. Issues in Comprehensive Pediatric Nursing. 1999;22(4):153-65.

137. Hustad JTP, Barnett NP, Borsari B, Jackson KM. Web-based alcohol prevention for incoming college students: a randomized controlled trial. Addictive Behaviors. 2010;35(3):183-9.

138. Iloabachie C, Wells C, Goodwin B, Baldwin M, Vanderplough-Booth K, Gladstone T, et al. Adolescent and parent experiences with a primary care/internet-based depression prevention intervention ((CATCH-IT). General Hospital Psychiatry. 2011;33(6):543-55.

139. Ip EJ, Lu DH, Barnett MJ, Tenerowicz MJ, Vo JC, Perry PJ. Psychological and physical impact of anabolic-androgenic steroid dependence. Pharmacotherapy:The Journal of Human Pharmacology & Drug Therapy. 2012;32(10):910-9.

140. Jackson JE. A survey of a Canadian on-line substance abuse prevention initiative for adolescents and young adults. Journal of Telemedicine & Telecare. 1995;1(4):217-23.

141. Jameson E, Trevena J, Swain N. Electronic gaming as pain distraction. Pain Research & Management. 2011;16(1):27-32.

142. Jenssen BP, Klein JD, Salazar LF, Daluga NA, DiClemente RJ. Exposure to tobacco on the internet: content analysis of adolescents' internet use. Pediatrics. 2009;124(2):e180-6.

143. Jones R, Sharkey S, Smithson J, Ford T, Emmens T, Hewis E, et al. Using metrics to describe the participative stances of members within discussion forums. Journal of Medical Internet Research. 2011;13(1):e3.

144. Jordan S. [[www.drugcom.de--an](http://www.drugcom.de--an) Internet based information and counselling project for the prevention of addiction]. Praxis der Kinderpsychologie und Kinderpsychiatrie. 2005;54(9):742-54.

145. Kanekar A, Sharma M, Atri A. Enhancing social support, hardiness, and acculturation to improve mental health among Asian Indian international students. International Quarterly of Community Health Education. 2009;30(1):55-68.

146. Kasatpibal N, Viseskul N, Srikantha W, Fongkaew W, Surapagdee N, Grimes RM. Developing a web site for human immunodeficiency virus prevention in a middle income country: a pilot study from Thailand. Cyberpsychology, behavior and social networking. 2012;15(10):560-3.

147. Kay-Lambkin FJ, Baker AL, Kelly B, Lewin TJ. Clinician-assisted computerised versus therapist-delivered treatment for depressive and addictive disorders: a randomised controlled trial. Medical Journal of Australia. 2011;195(3):S44-50.

148. Kelly BC, Carpiano RM, Easterbrook A, Parsons JT. Sex and the community: the implications of neighbourhoods and social networks for sexual risk behaviours among urban gay men. Sociology of Health & Illness. 2012;34(7):1085-102.

149. Kersting A, Kroker K, Schlicht S, Baust K, Wagner B. Efficacy of cognitive behavioral internet-based therapy in parents after the loss of a child during pregnancy: pilot data from a randomized controlled trial. Archives of Women's Mental Health. 2011;14(6):465-77.

150. Khanna MS, Kendall PC. Computer-assisted cognitive behavioral therapy for child anxiety: results of a randomized clinical trial. Journal of Consulting & Clinical Psychology. 2010;78(5):737-45.

151. Kim H, Stout PA. The Effects of Interactivity on Information Processing and Attitude Change: Implications for Mental Health Stigma. Health Communication. 2010;25(2):142-54.

152. King CA, Kramer A, Preuss L, Kerr DCR, Weisse L, Venkataraman S. Youth-Nominated Support Team for Suicidal Adolescents (Version 1): a randomized controlled trial. Journal of Consulting & Clinical Psychology. 2006;74(1):199-206.

153. Klein B, Meyer D, Austin DW, Kyrios M. Anxiety online: a virtual clinic: preliminary outcomes following completion of five fully automated treatment programs for anxiety disorders and symptoms. Journal of Medical Internet Research. 2011;13(4):e89.

154. Klein H. Sexual orientation, drug use preference during sex, and HIV risk practices and preferences among men who specifically seek unprotected sex partners via the internet. International Journal of Environmental Research & Public Health [Electronic Resource]. 2009;6(5):1620-35.

155. Klein JD, Havens CG, Carlson EJ. Evaluation of an adolescent smoking-cessation media campaign: GottaQuit.com. Pediatrics. 2005;116(4):950-6.

156. Ko C-H, Yen J-Y, Liua S-C, Huang C-F, Yen C-F. The associations between aggressive behaviors and internet addiction and online activities in adolescents. Journal of Adolescent Health. 2009;44(6):598-605.

157. Kokkonen R. The fat child—a sign of ‘bad’ motherhood? An analysis of explanations for children's fatness on a Finnish website. Journal of Community & Applied Social Psychology. 2009;19(5):336-47.

158. Kontos EZ, Emmons KM, Puleo E, Viswanath K. Communication inequalities and public health implications of adult social networking site use in the United States. Journal of Health Communication. 2010;15 Suppl 3:216-35.

159. Kowalski RM, Fedina C. Cyber Bullying in ADHD and Asperger Syndrome Populations. Research in Autism Spectrum Disorders. 1201;5(3):1201-8.

160. Kreitman N, Smith P, Tan ES. Attempted suicide in social networks. British Journal of Preventive & Social Medicine. 1969;23(2):116-23.

161. Kurki M, Koivunen M, Anttila M, Hatonen H, Valimaki M. Usefulness of internet in adolescent mental health outpatient care. Journal of Psychiatric & Mental Health Nursing. 2011;18(3):265-73.

162. Lane DJ, Lindemann DF, Schmidt JA. A comparison of computer-assisted and self-management programs for reducing alcohol use among students in first year experience courses. Journal of Drug Education. 2012;42(2):119-35.

163. Lange A, van de Ven JP, Schrieken B, Emmelkamp PM. Interapy, treatment of posttraumatic stress through the Internet: a controlled trial. Journal of Behavior Therapy & Experimental Psychiatry. 2001;32(2):73-90.

164. Lange JE, Daniel J, Homer K, Reed MB, Clapp JD. Salvia divinorum: effects and use among YouTube users. Drug & Alcohol Dependence. 2010;108(1-2):138-40.

165. Lariscy R, Reber B, Paek H-J. Exploration of health concerns and the role of social media information among rural and urban adolescents: a preliminary study. International Electronic Journal of Health Education. 2011;14:21p.

166. Lee CM, Atkins DC, Lewis MA, Kaysen D, Mittmann A, Fossos N, et al. A randomized controlled trial of event-specific prevention strategies for reducing problematic drinking associated with 21st birthday celebrations. Journal of Consulting & Clinical Psychology. 2012;80(5):850-62.

167. Lemola S, Brand S, Vogler N, Perkinson-Gloor N, Allemand M, Grob A. Habitual computer game playing at night is related to depressive symptoms. Personality and Individual Differences. 2011;51(2):117-22.

168. Leonard LG, Toller P. Speaking Ill of the Dead: Anonymity and Communication About Suicide on MyDeathSpace.com. Communication Studies. 2012;63(4):387-404.

169. Lester D. Bereavement after suicide: a study of memorials on the Internet. Omega - Journal of Death & Dying. 2012;65(3):189-94.

170. Leung SF, Ma J, Russell J. Breaking the silence of eating disorders with the hope of an online self-help programme. Contemporary Nurse. 2012;40(2):245-57.

171. Leung SFC, Russell J. An open trial of self-help behaviours of clients with eating disorders in an online programme. Journal of Advanced Nursing. 2013;69(1):66-76.

172. Lightfoot M, Comulada WS, Stover G. Computerized HIV preventive intervention for adolescents: indications of efficacy. American Journal of Public Health. 2007;97(6):1027-30.

173. Linke S, Murray E, Butler C, Wallace P. Internet-based interactive health intervention for the promotion of sensible drinking: patterns of use and potential impact on members of the general public. Journal of Medical Internet Research. 2007;9(2):e10.

174. Little L, Clark RR. Wonders and worries of parenting a child with Asperger syndrome & nonverbal learning disorder. MCN, American Journal of Maternal Child Nursing. 2006;31(1):39-44.

175. Livingstone S. Taking risky opportunities in youthful content creation: Teenagers' use of social networking sites for intimacy, privacy and self-expression. New Media & Society. 2008;10(3):393-411.

176. Locatelli SM, Kluwe K, Bryant FB. Facebook use and the tendency to ruminate among college students: testing mediational hypotheses. . Journal of Educational Computing Research. 2012;46(4):377-94.

177. Lord S, Brevard J, Budman S. Connecting to young adults: an online social network survey of beliefs and attitudes associated with prescription opioid misuse among college students.[Erratum appears in Subst Use Misuse.2011;46(4):560]. Substance Use & Misuse. 2011;46(1):66-76.

178. Lord SE, D'Amante D. Efficacy of online alcohol and other drug prevention for early adolescents. Journal of Adolescent Health. 2007;40(2):S4-S.

179. Lovecchio CP, Wyatt TM, DeJong W. Reductions in drinking and alcohol-related harms reported by first-year college students taking an online alcohol education course: a randomized trial. Journal of Health Communication. 2010;15(7):805-19.

180. Lwin MO, Miyazaki AD, Stanaland AJ, Lee E. Online usage motive and information disclosure for preteen children. Young Consumers. 2012;13(4):345-56.

181. Lynch M. Healthy Habits or Damaging Diets: An Exploratory Study of a Food Blogging Community. Ecology of Food and Nutrition. 2010;49(4):316-35.

182. Madell D, Muncer S. Internet communication: an activity that appeals to shy and socially phobic people? Cyberpsychology & Behavior. 2006;9(5):618-22.

183. Magee JC, Ritterband LM, Thorndike FP, Cox DJ, Borowitz SM. Exploring the relationship between parental worry about their children's health and usage of an internet intervention for pediatric encopresis. Journal of Pediatric Psychology. 2009;34(5):530-8.

184. Makarushka MM. Efficacy of an Internet-Based Intervention Targeted to Adolescents with Subthreshold Depression. ProQuest LLC PhD Dissertation, University of Oregon. 2011.

185. Makarushka MM. Efficacy of an Internet-based intervention targeted to adolescents with subthreshold depression. Dissertation Abstracts International Section A: Humanities and Social Sciences. 2012;73(3-A):977.

186. Malygin VL, Iskandirova AS, Khomeriki NS, Smirnova EA, Antonenko AA. [Personality features in adolescents with internet addiction]. Zhurnal Nevrologii i Psikhiatrii Imeni SS Korsakova. 2011;111(4):105-8.

187. March S, Spence SH, Donovan CL. The efficacy of an internet-based cognitive-behavioral therapy intervention for child anxiety disorders. Journal of Pediatric Psychology. 2009;34(5):474-87.

188. Marko M, Fogel J, Mykerezi E, Van Voorhees BW. Adolescent Internet depression prevention: Preferences for intervention and predictors of intentions and adherence. Journal of CyberTherapy and Rehabilitation. 2010;3(1):9-30.

189. Markovitzky O, Anholt GE, Lipsitz JD. Haven't we met somewhere before? The effects of a brief Internet introduction on social anxiety in a subsequent face to face interaction. Behaviour Research & Therapy. 2012;50(5):359-65.

190. Marsac ML, Kassam-Adams N, Hildenbrand AK, Kohser KL, Winston FK. After the injury: initial evaluation of a web-based intervention for parents of injured children. Health Education Research. 2011;26(1):1-12.

191. Marsch LA, Bickel WK, Badger GJ. Applying Computer Technology to Substance Abuse Prevention Science Results of a Preliminary Examination. Journal of Child and Adolescent Substance Abuse. 2007;16(2):69-94.

192. Marsch LA, Grabinski MJ, Bickel WK, Desrosiers A, Guarino H, Muehlbach B, et al. Computer-assisted HIV prevention for youth with substance use disorders. Substance Use & Misuse. 2011;46(1):46-56.

193. McCabe MP, Ricciardelli LA, Karantzas G. Impact of a healthy body image program among adolescent boys on body image, negative affect, and body change strategies. Body Image. 2010;7(2):117-23.

194. McFarlane M, Bull SS, Rietmeijer CA. Young adults on the Internet: risk behaviors for sexually transmitted diseases and HIV(1). Journal of Adolescent Health. 2002;31(1):11-6.

195. Mehta SA. What can physicians learn from the blogs of patients with uveitis? Ocular Immunology & Inflammation. 2007;15(6):421-3.

196. Merry S, Stasiak K, Shepherd M, Frampton C, Fleming T, Lucassen M. The effectiveness of SPARX, a computerised self help intervention for adolescents seeking help for depression: Randomised controlled non-inferiority trial. BMJ: British Medical Journal. 2012;344(7857):1-16.

197. Metcalf A, Blanchard M, McCarthy T, Burns J. Bridging the Digital Divide: Utilising technology to promote social connectedness and civic engagement amongst marginalised young people. 3CMedia: Journal of Community, Citizen's & Third Sector Media & Communication. 2008(4):2-15.

198. Mewton L, Wong N, Andrews G. The effectiveness of internet cognitive behavioural therapy for generalized anxiety disorder in clinical practice. Depression & Anxiety. 2012;29(10):843-9.

199. Meyerbroker K, Morina N, Kerkhof G, Emmelkamp PMG. Virtual reality exposure treatment of agoraphobia: a comparison of computer automatic virtual environment and head-mounted display. Studies in Health Technology & Informatics. 2011;167:51-6.

200. Michael K. Online sexual activities and sexual risk-taking among adolescents and young adults in Lagos Metropolis, Nigeria. African Journal of Reproductive Health. 2012;16(2):207-17.

201. Mieczynski ND. Cyberbullying: Exploring the experiences of mental health professionals. Dissertation Abstracts International: Section B: The Sciences and Engineering. 2009;70(6-B):3790.

202. Mikami AY, Szwedo DE, Allen JP, Evans MA, Hare AL. Adolescent Peer Relationships and Behavior Problems Predict Young Adults' Communication on Social Networking Websites. Developmental Psychology. 2010;46(1):46-56.

203. Mitchell KJ, Ybarra M, Finkelhor D. The relative importance of online victimization in understanding depression, delinquency, and substance use. Child Maltreatment. 2007;12(4):314-24.

204. Mitchell KJ, Finkelhor D, Wolak J, Ybarra ML, Turner H. Youth internet victimization in a broader victimization context. Journal of Adolescent Health. 2011;48(2):128-34.

205. Mitra R. Resisting the Spectacle of Pride: Queer Indian Bloggers as Interpretive Communities. Journal of Broadcasting & Electronic Media. 2010;54(1):163-78.

206. Mittal VA, Tessner KD, Walker EF. Elevated social Internet use and schizotypal personality disorder in adolescents. Schizophrenia Research. 2007;94(1-3):50-7.

207. Monks CP, Robinson S, Worlidge P. The Emergence of Cyberbullying: A Survey of Primary School Pupils' Perceptions and Experiences. School Psychology International. 2012;33(5):477-91.

208. Moreno MA, Parks M, Richardson LP. What are adolescents showing the world about their health risk behaviors on MySpace? Medgenmed [Computer File]: Medscape General Medicine. 2007;9(4):9.

209. Moreno MA, Briner LR, Williams A, Walker L, Christakis DA. Real Use or "Real Cool": Adolescents Speak Out About Displayed Alcohol References on Social Networking Websites. Journal of Adolescent Health. 2009;45(4):420-2.

210. Moreno MA, VanderStoep A, Parks MR, Zimmerman FJ, Kurth A, Christakis DA. Reducing at-risk adolescents' display of risk behavior on a social networking Web site: a randomized controlled pilot intervention trial. Archives of Pediatrics & Adolescent Medicine. 2009;163(1):35-41.

211. Moreno MA, Briner LR, Williams A, Brockman L, Walker L, Christakis DA. A content analysis of displayed alcohol references on a social networking web site. Journal of Adolescent Health. 2010;47(2):168-75.

212. Moreno MA, Grant A, Kacvinsky L, Egan KG, Fleming MF. College students' alcohol displays on Facebook: intervention considerations. Journal of American College Health. 2012;60(5):388-94.

213. Moritz S, Toews J, Rickhi B, Paccagnan P, Malhotra S, Hart C, et al. OA15.02. Quantitative findings from piloting the LEAP project: an online spirituality based depression intervention for young adults. BMC Complementary & Alternative Medicine. 2012;12(Suppl 1):1-.

214. Mottram AJ, Fleming MJ. Extraversion, impulsivity, and online group membership as predictors of problematic internet use. Cyberpsychology & Behavior. 2009;12(3):319-21.

215. Murray CD, Macdonald S, Fox J. Body satisfaction, eating disorders and suicide ideation in an Internet sample of self-harmers reporting and not reporting childhood sexual abuse. Psychology Health & Medicine. 2008;13(1):29-42.

216. Murray E, Kerr C, Stevenson F, Gore C, Nazareth I. Internet interventions can meet the emotional needs of patients and carers managing long-term conditions. Journal of Telemedicine & Telecare. 2007;13:42-4.

217. My H, Henry Y, Prue T. Mining data on usage of electronic nicotine delivery systems (ENDS) from YouTube videos. Tobacco Control. 2013;22(2):103-6.

218. Narhi U, Pohjanoksa-Mantyla M, Karjalainen A, Saari JK, Wahlroos H, Airaksinen MS, et al. The DARTS tool for assessing online medicines information. Pharmacy World & Science. 2008;30(6):898-906.

219. Navejas M, Neaigus A, Torian L, Murrill C. Participation in online and offline HIV prevention among men who have sex with men who use the internet to meet sex partners in New York City. AIDS & Behavior. 2012;16(2):389-95.

220. Nevonen L, Mark M, Levin B, Lindstrom M, Paulson-Karlsson G. Evaluation of a new Internet-based self-help guide for patients with bulimic symptoms in Sweden. Nordic Journal of Psychiatry. 2006;60(6):463-8.

221. Newman K, Powell V. Using videos, Vikings and teddy-bears to reduce anxiety: A methodology for implementing and evaluating fun experiences in the treatment of social anxiety disorders. Annual Review of CyberTherapy and Telemedicine. 2007;5:133-42.

222. Newton NC, Andrews G, Teesson M, Vogl LE. Delivering prevention for alcohol and cannabis using the Internet: a cluster randomised controlled trial. Preventive Medicine. 2009;48(6):579-84.

223. Newton NC, Teesson M, Vogl LE, Andrews G. Internet-based prevention for alcohol and cannabis use: final results of the Climate Schools course. Addiction. 2010;105(4):749-59.

224. Nicholas J. The role of internet technology and social branding in improving the mental health and wellbeing of young people. Perspectives in Public Health. 2010;130(2):86-90.

225. Norman CD, Maley O, Li X, Skinner HA. Using the Internet to assist smoking prevention and cessation in schools: a randomized, controlled trial. Health Psychology. 2008;27(6):799-810.

226. Nygaard P, Paschall MJ. Students' experiences with web-based alcohol prevention: a qualitative evaluation of AlcoholEdu. Journal of Drug Education. 2012;42(2):137-58.

227. O'Dea B, Campbell A. Online social networking amongst teens: friend or foe? Studies in Health Technology & Informatics. 2011;167:133-8.

228. O'Dea B, Campbell A. Healthy connections: online social networks and their potential for peer support. Studies in Health Technology & Informatics. 2011;168:133-40.

229. O'Kearney R, Gibson M, Christensen H, Griffiths KM. Effects of a cognitive-behavioural internet program on depression, vulnerability to depression and stigma in adolescent males: A school based controlled trial. Australian Journal of Psychology. 2005;57:238-9.

230. O'Kearney R, Kang K, Christensen H, Griffiths K. A controlled trial of a school-based Internet program for reducing depressive symptoms in adolescent girls. Depression and anxiety. 2009;26(1):65-72.

231. Okamoto J. Social network influences on depressive symptoms among Chinese adolescents. Dissertation Abstracts International: Section B: The Sciences and Engineering. 2011;71(9-B):5413.

232. Olenik-Shemesh D, Heiman T, Eden S. Cyberbullying Victimisation in Adolescence: Relationships with Loneliness and Depressive Mood. Emotional and Behavioural Difficulties. 2012;17(3):361-74.

233. Owen JE, Boxley L, Goldstein MS, Lee JH, Breen N, Rowland JH. Use of Health-Related Online Support Groups: Population Data from the California Health Interview Survey Complementary and Alternative Medicine Study. Journal of Computer-Mediated Communication. 2010;15(3):427-46.

234. Pacifici C, Delaney R, White L, Cummings K, Nelson C. Foster Parent College: Interactive Multimedia Training for Foster Parents. Social Work Research. 2005;29(4):243.

235. Padgett LS, Strickland D, Coles CD. Case study: using a virtual reality computer game to teach fire safety skills to children diagnosed with fetal alcohol syndrome. Journal of Pediatric Psychology. 2006;31(1):65-70.

236. Palermo TM, Wilson AC, Peters M, Lewandowski A, Somhegyi H. Randomized controlled trial of an Internet-delivered family cognitive-behavioral therapy intervention for children and adolescents with chronic pain. Pain (03043959). 2009;146(1-2):205-13.

237. Pantic I, Damjanovic A, Todorovic J, Topalovic D, Bojovic-Jovic D, Ristic S, et al. Association between online social networking and depression in high school students: behavioral physiology viewpoint. Psychiatria Danubina. 2012;24(1):90-3.

238. Park K-M, Ku J, Choi S-H, Jang H-J, Park J-Y, Kim SI, et al. A virtual reality application in role-plays of social skills training for schizophrenia: a randomized, controlled trial. Psychiatry Research. 2011;189(2):166-72.

239. Parkinson AB, Evans NA. Anabolic androgenic steroids: a survey of 500 users. Medicine & Science in Sports & Exercise. 2006;38(4):644-51.

240. Parlove AE, Cowdery JE, Hoerauf SL. Acceptability and appeal of a Web-based smoking prevention intervention for adolescents. International Electronic Journal of Health Education. 2004;7:1-8.

241. Parsons TD, Bowerly T, Buckwalter JG, Rizzo AA. A controlled clinical comparison of attention performance in children with ADHD in a virtual reality classroom compared to standard neuropsychological methods. Child Neuropsychology. 2007;13(4):363-81.

242. Parsons TD, Kenny P, Cosand L, Iyer A, Courtney C, Rizzo AA. A virtual human agent for assessing bias in novice therapists. Studies in Health Technology & Informatics. 2009;142:253-8.

243. Peebles R, Wilson JL, Litt IF, Hardy KK, Lock JD, Mann JR, et al. Disordered eating in a digital age: eating behaviors, health, and quality of life in users of websites with pro-eating disorder content. Journal of Medical Internet Research. 2012;14(5):e148.

244. Pemberton MR, Williams J, Herman-Stahl M, Calvin SL, Bradshaw MR, Bray RM, et al. Evaluation of two web-based alcohol interventions in the U.S. military. Journal of Studies on Alcohol & Drugs. 2011;72(3):480-9.

245. Pinto MD, Hickman RL, Clochesy J, Buchner M. Avatar-based depression self-management technology: promising approach to improve depressive symptoms among young adults... Self-Management Resource Training for Mental Health (eSMART-MH). Applied Nursing Research. 2013;26(1):45-8.

246. Pittaway S, Cupitt C, Palmer D, Arowobusoye N, Milne R, Holttum S, et al. Comparative, clinical feasibility study of three tools for delivery of cognitive behavioural therapy for mild to moderate depression and anxiety provided on a self-help basis. Mental Health in Family Medicine. 2009;6(3):145-54.

247. Postel MG, de Haan HA, ter Huurne ED, van der Palen J, Becker ES, de Jong CAJ. Attrition in web-based treatment for problem drinkers. Journal of Medical Internet Research. 2011;13(4):e117.

248. Pretlow RA. Addiction to highly pleasurable food as a cause of the childhood obesity epidemic: a qualitative Internet study. Brunner-Mazel Eating Disorders Monograph Series. 2011;19(4):295-307.

249. Price J, Cole V, Goodwin GM. Emotional side-effects of selective serotonin reuptake inhibitors: qualitative study. British Journal of Psychiatry. 2009;195(3):211-7.

250. Priebe G, Svedin CG. Online or off-line victimisation and psychological well-being: a comparison of sexual-minority and heterosexual youth. European Child & Adolescent Psychiatry. 2012;21(10):569-82.

251. Prokhorov AV, Kelder SH, Shegog R, Murray N, Peters R, Jr., Agurcia-Parker C, et al. Impact of A Smoking Prevention Interactive Experience (ASPIRE), an interactive, multimedia smoking prevention and cessation curriculum for culturally diverse high-school students. Nicotine & Tobacco Research. 2008;10(9):1477-85.

252. Proudfoot JG, Parker GB, Benoit M, Manicavasagar V, Smith M, Gayed A. What happens after diagnosis? Understanding the experiences of patients with newly-diagnosed bipolar disorder. Health Expectations. 2009;12(2):120-9.

253. Pujazon-Zazik MA, Manasse SM, Orrell-Valente JK. Adolescents' self-presentation on a teen dating web site: A risk-content analysis. Journal of Adolescent Health. 2012;50(5):517-20.

254. Radhu N, Daskalakis ZJ, Arpin-Cribbie CA, Irvine JR, Paul. Evaluating a Web-based cognitive-behavioral therapy for maladaptive perfectionism in university students. Journal of American College Health. 2012;60(5):357-66.

255. Ragatz LL, Anderson RJ, Fremouw W, Schwartz R. Criminal thinking patterns, aggression styles, and the psychopathic traits of late high school bullies and bully-victims. Aggressive Behavior. 2011;37(2):145-60.

256. Ramo DE, Prochaska JJ. Broad reach and targeted recruitment using Facebook for an online survey of young adult substance use. Journal of Medical Internet Research. 2012;14(1):e28.

257. Raskind MH, Margalit M, Higgins EL. "My LD": Children's Voices on the Internet. Learning Disability Quarterly. 2006;29(4):253-68.

258. Reavley NJ, Morgan AJ, Jorm D, Jorm AF. An Evaluation of Mental Health Wiki: A Consumer Guide to Mental Health Information on the Internet. Journal of Consumer Health on the Internet. 2013;17(1):1-9.

259. Remondino G. Blog y redes sociales: un análisis desde las tecnologías de la gubernamentalidad y el género. (Spanish). Blog and social networks: an analysis from the governmentality technologies and gender (English). 2012;12(3):51-69.

260. Renner B, Schmalzle R, Schupp HT. First impressions of HIV risk: it takes only milliseconds to scan a stranger. PLoS ONE [Electronic Resource]. 2012;7(1):e30460.

261. Reupert A, Goodyear M, Eddy K, Alliston C, Mason P, Maybery D, et al. Australian programs and workforce initiatives for children and their families where a parent has a mental illness. AeJAMH (Australian e-Journal for the Advancement of Mental Health). 2009;8(3):277-85.

262. Reupert A, Foster K, Maybery D, Eddy K, Fudge E. 'Keeping families and children in mind': An evaluation of a web-based workforce resource. Child & Family Social Work. 2011;16(2):192-200.

263. Reupert AE, Maybery DJ. A "snapshot" of Australian programs to support children and adolescents whose parents have a mental illness. Psychiatric Rehabilitation Journal. 2009;33(2):125-32.

264. Rice E. The positive role of social networks and social networking technology in the condom-using behaviors of homeless young people. Public Health Reports. 2010;125(4):588-95.

265. Rice E, Milburn NG, Monro W. Social networking technology, social network composition, and reductions in substance use among homeless adolescents. Prevention Science. 2011;12(1):80-8.

266. Rice E, Kurzban S, Ray D. Homeless but connected: the role of heterogeneous social network ties and social networking technology in the mental health outcomes of street-living adolescents. Community Mental Health Journal. 2012;48(6):692-8.

267. Richards D, Timulak L. Satisfaction with therapist-delivered vs. self-administered online cognitive behavioural treatments for depression symptoms in college students. British Journal of Guidance & Counselling. 2013;41(2):193-207.

268. Richards JC, Klein B, Austin DW. Internet cognitive behavioural therapy for panic disorder: does the inclusion of stress management information improve end-state functioning? Clinical Psychologist. 2006;10(1):2-15.

269. Rizzo AS, Difede J, Rothbaum BO, Reger G, Spitalnick J, Cukor J, et al. Development and early evaluation of the Virtual Iraq/Afghanistan exposure therapy system for combat-related PTSD. Annals of the New York Academy of Sciences. 2010;1208:114-25.

270. Robinson E, Titov N, Andrews G, McIntyre K, Schwencke G, Solley K. Internet treatment for generalized anxiety disorder: a randomized controlled trial comparing clinician vs. technician assistance. PLoS ONE. 2010;5(6):e10942.

271. Rodgers J, Buchanan T, Pearson C, Parrott AC, Ling J, Hefferman TM, et al. Differential experiences of the psychobiological sequelae of ecstasy use: quantitative and qualitative data from an internet study. Journal of Psychopharmacology. 2006;20(3):437-46.

272. Rosen LD, Whaling K, Rab S, Carrier LM, Cheever NA. Is Facebook creating “iDisorders”? The link between clinical symptoms of psychiatric disorders and technology use, attitudes and anxiety. Computers in Human Behavior. 2013;29(3):1243-54.

273. Rosser BRS, Oakes JM, Konstan J, Hooper S, Horvath KJ, Danilenko GP, et al. Reducing HIV risk behavior of men who have sex with men through persuasive computing: results of the Men's INTernet Study-II. AIDS. 2010;24(13):2099-107.

274. Ruggiero KJ, Resnick HS, Acierno R, Coffey SF, Carpenter MJ, Ruscio AM, et al. Internet-based intervention for mental health and substance use problems in disaster-affected populations: a pilot feasibility study. Behavior Therapy. 2006;37(2):190-205.

275. Rushing SC, Stephens D. Use of Media Technologies by Native American Teens and Young Adults in the Pacific Northwest: Exploring Their Utility for Designing Culturally Appropriate Technology-Based Health Interventions. Journal of Primary Prevention. 2011;32(3):135-45.

276. Ruwaard J, Lange A, Schrieken B, Dolan CV, Emmelkamp P. The effectiveness of online cognitive behavioral treatment in routine clinical practice. PLoS ONE [Electronic Resource]. 2012;7(7):e40089.

277. Sanders MR, Baker S, Turner KMT. A randomized controlled trial evaluating the efficacy of Triple P Online with parents of children with early-onset conduct problems. Behaviour Research & Therapy. 2012;50(11):675-84.

278. Saulsberry A, Corden M, Taylor-Crawford K, Crawford T, Johnson M, Froemel J, et al. Chicago Urban Resiliency Building (CURB): An Internet-Based Depression-Prevention Intervention for Urban African-American and Latino Adolescents. Journal of Child & Family Studies. 2013;22(1):150-60.

279. Saylor CF, Cowart BL, Lipovsky JA, Jackson C, Finch A. Media exposure to September 11: Elementary school students' experiences and posttraumatic symptoms. American Behavioral Scientist. 2003;46(12):1622-42.

280. Scharer K, Colon E, Moneyham L, Hussey J, Tavakoli A, Shugart M. A comparison of two types of social support for mothers of mentally ill children. Journal of Child & Adolescent Psychiatric Nursing. 2009;22(2):86-98.

281. Schinke SP, Fang L, Cole KC. Computer-delivered, parent-involvement intervention to prevent substance use among adolescent girls. Preventive Medicine. 2009;49(5):429-35.

282. Schmidt K. What Works: The Results of Evaluations on Two Interactive Multimedia Programs. International Journal of Instructional Media. 1994;21(4):269-77.

283. Schneider SK, O'Donnell L, Stueve A, Coulter RWS. Cyberbullying, school bullying, and psychological distress: a regional census of high school students. American Journal of Public Health. 2012;102(1):171-7.

284. Schoech D. Developing a virtual community to prevent teen substance abuse: Lessons learned. Journal of Technology in Human Services. 2007;25(3):81-100.

285. Scholle SH, Peele PB, Kelleher KJ, Frank E, Jansen-McWilliams L, Kupfer D. Effect of different recruitment sources on the composition of a bipolar disorder case registry. Social Psychiatry and Psychiatric Epidemiology. 2000;35(5):220-7.

286. Schuckit MA, Kalmijn JA, Smith TL, Saunders G, Fromme K. Structuring a College Alcohol Prevention Program on the Low Level of Response to Alcohol Model: A Pilot Study. Alcoholism: Clinical & Experimental Research. 2012;36(7):1244-52.

287. Schultze-Krumbholz A, Jakel A, Schultze M, Scheithauer H. Emotional and Behavioural Problems in the Context of Cyberbullying: A Longitudinal Study among German Adolescents. Emotional and Behavioural Difficulties. 2012;17(3):329-45.

288. Schwinn TM. Substance use among late adolescent urban youth: Correlates and longitudinal outcomes. Dissertation Abstracts International Section A: Humanities and Social Sciences. 2010;70(8-A):3195.

289. Seepersad SS. Understanding and helping the lonely: An evaluation of the LUV program. Dissertation Abstracts International: Section B: The Sciences and Engineering. 2006;66(7-B):4002.

290. Seidman DF, Westmaas JL, Goldband S, Rabius V, Katkin ES, Pike KJ, et al. Randomized controlled trial of an interactive internet smoking cessation program with long-term follow-up. Annals of Behavioral Medicine. 2010;39(1):48-60.

291. Selfhout MHW, Branje SJT, Delsing M, ter Bogt TFM, Meeus WHJ. Different types of Internet use, depression, and social anxiety: the role of perceived friendship quality. Journal of Adolescence. 2009;32(4):819-33.

292. Sethi S, Campbell AJ, Ellis LA. The use of computerized self-help packages to treat adolescent depression and anxiety. Journal of Technology in Human Services. 2010;28(3):144-60.

293. Shandley K, Klein B, Austin D. The players' perspective of Reach Out Central: A therapeutic interactive online game. E-Journal of Applied Psychology. 2008;4(2):51-5.

294. Shapira LB, Mongrain M. The benefits of self-compassion and optimism exercises for individuals vulnerable to depression. Journal of Positive Psychology. 2010;5(5):377-89.

295. Sherman LE, Greenfield PM. Forging friendship, soliciting support: A mixed-method examination of message boards for pregnant teens and teen mothers. Computers in Human Behavior. 2013;29(1):75-85.

296. Silenzio VMB, Duberstein PR, Tang W, Lu N, Tu X, Homan CM. Connecting the invisible dots: reaching lesbian, gay, and bisexual adolescents and young adults at risk for suicide through online social networks. Social Science & Medicine. 2009;69(3):469-74.

297. Sinclair KO, Bauman S, Poteat V, Koenig B, Russell ST. Cyber and bias-based harassment: Associations with academic, substance use, and mental health problems. Journal of Adolescent Health. 2012;50(5):521-3.

298. Sisask M, Varnik A, Wasserman D. Internet comments on media reporting of two adolescents' collective suicide attempt. Archives of Suicide Research. 2005;9(1):87-98.

299. Smith-Simone S, Maziak W, Ward KD, Eissenberg T. Waterpipe tobacco smoking: knowledge, attitudes, beliefs, and behavior in two U.S. samples. Nicotine & Tobacco Research. 2008;10(2):393-8.

300. Smith LE, Greenberg JS, Seltzer MM. Social support and well-being at mid-life among mothers of adolescents and adults with autism spectrum disorders. Journal of Autism & Developmental Disorders. 2012;42(9):1818-26.

301. Smith PK, Mahdavi J, Carvalho M, Fisher S, Russell S, Tippett N. Cyberbullying: its nature and impact in secondary school pupils. Journal of Child Psychology & Psychiatry. 2008;49(4):376-85.

302. Snodgrass JG, Lacy MG, Francois Dengah HJ, 2nd, Fagan J, Most DE. Magical flight and monstrous stress: technologies of absorption and mental wellness in Azeroth.[Erratum appears in Cult Med Psychiatry. 2011 Sep;35(3):446]. Culture, Medicine & Psychiatry. 2011;35(1):26-62.

303. Soutter J, Hamilton N, Russell P, Russell C, Bushby K, Sloper P, et al. The Golden Freeway: a preliminary evaluation of a pilot study advancing information technology as a social intervention for boys with Duchenne muscular dystrophy and their families. Health & Social Care in the Community. 2004;12(1):25-33.

304. Spijkerman R, Roek MAE, Vermulst A, Lemmers L, Huiberts A, Engels RCME. Effectiveness of a web-based brief alcohol intervention and added value of normative feedback in reducing underage drinking: a randomized controlled trial. Journal of Medical Internet Research. 2010;12(5):e65.

305. Spiranovic C, Briggs K, Kirkby K, Mobsby C, Daniels B. Yshareit: A Project Promoting the Use of E-Mental Health Resources among Young People. Youth Studies Australia. 2008;27(2):52-60.

306. Spring B, Schneider K, McFadden HG, Vaughn J, Kozak AT, Smith M, et al. Multiple Behavior Changes in Diet and Activity: A Randomized Controlled Trial Using Mobile TechnologyBehavior Changes in Diet and Activity. Archives of internal medicine. 2012;172(10):789-96.

307. St-Jacques J, Bouchard S, Belanger C. Is virtual reality effective to motivate and raise interest in phobic children toward therapy? A clinical trial study of in vivo with in virtuo versus in vivo only treatment exposure. Journal of Clinical Psychiatry. 2010;71(7):924-31.

308. Stahl C, Fritz N. Internet safety: adolescents' self-report. Journal of Adolescent Health. 2002;31(1):7-10.

309. Stein K, Dyer M, Crabb T, Milne R, Round A, Ratcliffe J, et al. A pilot Internet "value of health" panel: recruitment, participation and compliance. Health & Quality of Life Outcomes. 2006;4:90.

310. Steinberger CB. Cyberspace: the nodal self in the wide wide world-adolescents signing-on. Psychoanalytic Review. 2009;96(1):129-44.

311. Stevkovski G. Shyness and its relationship to body dissatisfaction, disordered eating, and thin-ideal internalization. Dissertation Abstracts International: Section B: The Sciences and Engineering. 2010;70(7-B):4498.

312. Stice E, Rohde P, Durant S, Shaw H. A preliminary trial of a prototype Internet dissonance-based eating disorder prevention program for young women with body image concerns. Journal of Consulting & Clinical Psychology. 2012;80(5):907-16.

313. Stommel W. Mein Nick bin ich! Nicknames in a German forum on eating disorders. Journal of Computer-Mediated Communication. 2007;13(1):141-62.

314. Strecher VJ, Shiffman S, West R. Moderators and mediators of a web-based computer-tailored smoking cessation program among nicotine patch users. Nicotine & Tobacco Research. 2006;8 Suppl 1:S95-101.

315. Struik LL, Bottorff JL, Jung M, Budgen C. Reaching Adolescent Girls Through Social Networking: A New Avenue for Smoking Prevention Messages. Canadian Journal of Nursing Research. 2012;44(3):84-103.

316. Swannell S, Oam GM, Krysinska K, Kay T, Olsson K, Win A. Cutting on-line: Self-injury and the internet. Advances in Mental Health. 2010;9(2):177-89.

317. Syed-Abdul S, Fernandez-Luque L, Jian WS, Li YC, Crain S, Hsu MH, et al. Misleading Health-Related Information Promoted Through Video-Based Social Media: Anorexia on YouTube. Journal of Medical Internet Research. 2013;15(2).

318. Szwedo DE, Mikami AY, Allen JP. Qualities of Peer Relations on Social Networking Websites: Predictions from Negative Mother-Teen Interactions. Journal of Research on Adolescence. 2011;21(3):595-607.

319. Szwedo DE, Mikami AY, Allen JP. Social Networking Site Use Predicts Changes in Young Adults' Psychological Adjustment. Journal of Research on Adolescence. 2012;22(3):453-66.

320. Tahiroglu AY, Celik GG, Fettahoglu C, Yildirim V, Toros F, Avci A, et al. Problematic Internet use in the psychiatric sample compared community sample. Noropsikiyatri Arsivi / Archives of Neuropsychiatry. 2010;47(3):241-6.

321. Tang S, Tian L, Cao WW, Zhang K, Detels R, Li VC. Improving Reproductive Health Knowledge in Rural China—A Web-Based Strategy. Journal of Health Communication. 2009;14(7):690-714.

322. te Wildt BT, Putzig I, Drews M, Lampen-Imkamp S, Zedler M, Wiese B, et al. Pathological Internet use and psychiatric disorders: A cross-sectional study on psychiatric phenomenology and clinical relevance of Internet dependency. The European Journal of Psychiatry. 2010;24(3):136-45.

323. Thomée S, Härenstam A, Hagberg M. Computer use and stress, sleep disturbances, and symptoms of depression among young adults - a prospective cohort study. BMC Psychiatry. 2012;12(1):176-89.

324. Thompson SH, Lougheed E. Frazzled by Facebook? An Exploratory Study of Gender Differences in Social Network Communication among Undergraduate Men and Women. College Student Journal. 2012;46(1):88-98.

325. Tossmann H-P, Jonas B, Tensil M-D, Lang P, Struber E. A controlled trial of an internet-based intervention program for cannabis users. Cyberpsychology, behavior and social networking. 2011;14(11):673-9.

326. Townsend L, Gearing RE, Polyanskaya O. Influence of health beliefs and stigma on choosing internet support groups over formal mental health services. Psychiatric Services. 2012;63(4):370-6.

327. Tsouvelas G, Giotakos O. [Internet use and pathological internet engagement in a sample of college students]. Psychiatriki. 2011;22(3):221-30.

328. Ullman SE. Comparing gang and individual rapes in a community sample of urban women. Violence & Victims. 2007;22(1):43-51.

329. Valcke M, De Wever B, Van Keer H, Schellens T. Long-term study of safe internet use of young children. Computers & Education. 2011;57(1):1292-305.

330. Valente TW, Zogg JB, Christensen S, Richardson J, Kovacs A, Operskalski E. Using social networks to recruit an HIV vaccine preparedness cohort. Journal of Acquired Immune Deficiency Syndromes: JAIDS. 2009;52(4):514-23.

331. van der Zanden R, Kramer J, Gerrits R, Cuijpers P. Effectiveness of an online group course for depression in adolescents and young adults: A randomized trial. Journal of Medical Internet Research. 2012;14(3):296-309.

332. van Hoof JJ, Mulder J, Korte J, Postel MG, Pieterse ME. Dutch adolescent private drinking places: prevalence, alcohol consumption, and other risk behaviors. Alcohol. 2012;46(7):687-93.

333. van Rooij AJ, Schoenmakers TM. Compulsive Internet use: the role of online gaming and other Internet applications. Journal of Adolescent Health. 2010;47(1):51-7.

334. Van Voorhees BW, Ellis J, Stuart S, Fogel J, Ford DE. Pilot Study of a Primary Care Internet-Based Depression Prevention Intervention for Late Adolescents. Canadian Child and Adolescent Psychiatry Review. 2005;14(2):40-3.

335. Van Voorhees BW, Ellis JM, Gollan JK, Bell CC, Stuart SS, Fogel J, et al. Development and process evaluation of a primary care Internet-based intervention to prevent depression in emerging adults. Primary Care Companion to the Journal of Clinical Psychiatry. 2007;9(5):346-55.

336. Van Voorhees BW, Vanderplough-Booth K, Fogel J, Gladstone T, Bell C, Stuart S, et al. Integrative Internet-based depression prevention for adolescents: A randomized clinical trial in primary care for vulnerability and protective factors. Journal of the Canadian Academy of Child and Adolescent Psychiatry / Journal de l'Academie canadienne de psychiatrie de l'enfant et de l'adolescent. 2008;17(4):184-96.

337. Van Voorhees BW, Fogel J, Reinecke MA, Gladstone T, Stuart S, Gollan J, et al. Randomized clinical trial of an Internet-based depression prevention program for adolescents (Project CATCH-IT) in primary care: 12-week outcomes. Journal of Developmental & Behavioral Pediatrics. 2009;30(1):23-37.

338. Van Zalk MH, Branje SJ, Denissen J, Van Aken MA, Meeus WH. Who benefits from chatting, and why?: the roles of extraversion and supportiveness in online chatting and emotional adjustment. Personality & Social Psychology Bulletin. 2011;37(9):1202-15.

339. Vandemark NR, Burrell NR, Lamendola WF, Hoich CA, Berg NP, Medina E. An exploratory study of engagement in a technology-supported substance abuse intervention. Substance Abuse Treatment, Prevention & Policy. 2010;5(1):10-.

340. Veinot TC, Campbell TR, Kruger D, Grodzinski A, Franzen S. Drama and danger: the opportunities and challenges of promoting youth sexual health through online social networks. AMIA Annual Symposium Proceedings/AMIA Symposium. 2011;2011:1436-45.

341. Vilhelmsson A, Svensson T, Meeuwisse A, Carlsten A. What can we learn from consumer reports on psychiatric adverse drug reactions with antidepressant medication? Experiences from reports to a consumer association. BMC Clinical Pharmacology. 2011;11:16.

342. Vita A, De Peri L, Barlati S, Cacciani P, Deste G, Poli R, et al. Effectiveness of different modalities of cognitive remediation on symptomatological, neuropsychological, and functional outcome domains in schizophrenia: A prospective study in a real-world setting. Schizophrenia Research. 2011;133(1-3):223-31.

343. Vyas AN, Landry M, Schnider M, Rojas AM, Wood SF. Public health interventions: Reaching Latino adolescents via short message service and social media. Journal of Medical Internet Research. 2012;14(4):31-40.

344. Wade SL, Carey J, Wolfe CR. An online family intervention to reduce parental distress following pediatric brain injury. Journal of Consulting & Clinical Psychology. 2006;74(3):445-54.

345. Wade SL, Walz NC, Carey JC, Williams KM. Preliminary efficacy of a Web-based family problem-solving treatment program for adolescents with traumatic brain injury. Journal of Head Trauma Rehabilitation. 2008;23(6):369-77.

346. Wade SL, Walz NC, Carey J, McMullen KM, Cass J, Mark E, et al. Effect on behavior problems of teen online problem-solving for adolescent traumatic brain injury. Pediatrics. 2011;128(4):e947-53.

347. Wade SL, Walz NC, Carey J, McMullen KM, Cass J, Mark E, et al. A Randomized Trial of Teen Online Problem Solving: Efficacy in Improving Caregiver Outcomes After Brain Injury. Health Psychology. 2012;31(6):767-76.

348. Wade TD, Davidson S, O'Dea JA. A preliminary controlled evaluation of a school-based media literacy program and self-esteem program for reducing eating disorder risk factors. The International journal of eating disorders. 2003;33(4):371-83; discussion 84-7.

349. Walrave M, Heirman W. Cyberbullying: Predicting Victimisation and Perpetration. Children and Society. 2011;25(1):59-72.

350. Walther JB, DeAndrea D, Kim J, Anthony JC. The Influence of Online Comments on Perceptions of Antimarijuana Public Service Announcements on YouTube. Human Communication Research. 2010;36(4):469-92.

351. Wang H, Zhou X, Lu C, Wu J, Deng X, Hong L, et al. Adolescent bullying involvement and psychosocial aspects of family and school life: a cross-sectional study from Guangdong Province in China. PLoS ONE [Electronic Resource]. 2012;7(7):e38619.

352. Wang J, Iannotti RJ, Luk JW, Nansel TR. Co-occurrence of victimization from five subtypes of bullying: physical, verbal, social exclusion, spreading rumors, and cyber. Journal of Pediatric Psychology. 2010;35(10):1103-12.

353. Weaver JB, 3rd, Mays D, Sargent Weaver S, Kannenberg W, Hopkins GL, Eroglu D, et al. Health-risk correlates of video-game playing among adults. American Journal of Preventive Medicine. 2009;37(4):299-305.

354. Welker HS. Principal Perspectives on Social Networking and the Disruptive Effects of Cyberbullying. ProQuest LLC PhD Dissertation, Walden University. 2010.

355. Wentz E, Nyden A, Krevers B. Development of an internet-based support and coaching model for adolescents and young adults with ADHD and autism spectrum disorders: A pilot study. European Child & Adolescent Psychiatry. 2012;21(11):611-22.

356. Wetter DW, McClure JB, Cofta-Woerpel L, Costello TJ, Reitzel LR, Businelle MS, et al. A randomized clinical trial of a palmtop computer-delivered treatment for smoking relapse prevention among women. Psychology of Addictive Behaviors. 2011;25(2):365-71.

357. Whitehill JM, Brockman LN, Moreno MA. “Just Talk to Me”: Communicating With College Students About Depression Disclosures on Facebook. Journal of Adolescent Health. 2013;52(1):122-7.

358. Wilkerson JM, Smolenski DJ, Horvath KJ, Danilenko GP, Simon Rosser BR. Online and offline sexual health-seeking patterns of HIV-negative men who have sex with men. AIDS & Behavior. 2010;14(6):1362-70.

359. Wilkerson JM, Danilenko GP, Smolenski DJ, Myer BB, Rosser BRS. The role of critical self-reflection of assumptions in an online HIV intervention for men who have sex with men. AIDS Education & Prevention. 2011;23(1):13-24.

360. Williams AL, Merten MJ. A review of online social networking profiles by adolescents: implications for future research and intervention. Adolescence. 2008;43(170):253-74.

361. Williams S, Reid M. 'It's like there are two people in my head': a phenomenological exploration of anorexia nervosa and its relationship to the self. Psychology & Health. 2012;27(7):798-815.

362. Wilsie CC, Brestan-Knight E. Using an online viewing system for Parent-Child Interaction therapy consulting with professionals. Psychological Services. 2012;9(2):224-6.

363. Wisner KL, Logsdon MC, Shanahan BR. Web-based education for postpartum depression: conceptual development and impact. Archives of Women's Mental Health. 2008;11(5-6):377-85.

364. Wood RTA, Griffiths MD. Online guidance, advice, and support for problem gamblers and concerned relatives and friends: an evaluation of the GamAid pilot service. British Journal of Guidance & Counselling. 2007;35(4):373-89.

365. Woodruff SI, Conway TL, Edwards CC. Sociodemographic and smoking-related psychosocial predictors of smoking behavior change among high school smokers. Addictive Behaviors. 2008;33(2):354-8.

366. Wright A, McGorry PD, Harris MG, Jorm AF, Pennell K. Development and evaluation of a youth mental health community awareness campaign - The Compass Strategy. BMC Public Health. 2006;6:215.

367. Xie Y-B, Peng Z-W, Xu L-P. Association between Internet addiction and self-injurious behavior among middle school students in Guangzhou City. Chinese Mental Health Journal. 2010;24(6):469-72.

368. Ybarra ML, Mitchell KJ. Online Aggressor/Targets, Aggressors, and Targets: A Comparison of Associated Youth Characteristics. Journal of Child Psychology and Psychiatry. 1308;45(7):1308-16.

369. Ybarra ML. Linkages between depressive symptomatology and Internet harassment among young regular Internet users. Cyberpsychology & Behavior. 2004;7(2):247-57.

370. Ybarra ML, Leaf PJ, Diener-West M. Sex differences in youth-reported depressive symptomatology and unwanted internet sexual solicitation. Journal of Medical Internet Research. 2004;6(1):e5.

371. Ybarra ML, Mitchell KJ. Youth engaging in online harassment: associations with caregiver-child relationships, Internet use, and personal characteristics. Journal of Adolescence. 2004;27(3):319-36.

372. Ybarra ML, Alexander C, Mitchell KJ. Depressive symptomatology, youth Internet use, and online interactions: A national survey.[Erratum appears in J Adolesc Health. 2006 Jan;38(1):92]. Journal of Adolescent Health. 2005;36(1):9-18.

373. Ybarra ML, Diener-West M, Leaf PJ. Examining the overlap in Internet harassment and school bullying: implications for school intervention. Journal of Adolescent Health. 2007;41(6):S42-50.

374. Ybarra ML, Mitchell KJ, Finkelhor D, Wolak J. Internet prevention messages: targeting the right online behaviors. Archives of Pediatrics & Adolescent Medicine. 2007;161(2):138-45.

375. Yellowlees PM, Cook JN. Education about hallucinations using an internet virtual reality system: a qualitative survey. Academic Psychiatry. 2006;30(6):534-9.

376. Yellowlees PM, Hilty DM, Marks SL, Neufeld J, Bourgeois JA. A Retrospective Analysis of a Child and Adolescent eMental Health Program. Journal of the American Academy of Child and Adolescent Psychiatry. 2008;47(1):103.

377. Yoshii H, Watanabe Y, Kitamura H, Chen J, Akazawa K. Effect of an education program on improving knowledge of schizophrenia among parents of junior and senior high school students in Japan. BMC Public Health. 2011;11:323.

378. Yoshii H, Watanabe Y, Kitamura H, Nan Z, Akazawa K. Effect of an education program on improving help-seeking among parents of junior and senior high school students in Japan. Global Journal of Health Science. 2012;4(1):33-41.

379. Young M, Richards C, Gunning M. Online mental health resources for teenagers: An evaluation of two websites developed for adolescents. Advances in School Mental Health Promotion. 2012;5(4):277-89.

380. Young SD, Rice E. Online social networking technologies, HIV knowledge, and sexual risk and testing behaviors among homeless youth. AIDS & Behavior. 2011;15(2):253-60.

381. Zimmerman DP. A Psychosocial Comparison of Computer-Mediated and Face-to-Face Language Use among Severely Disturbed Adolescents. Adolescence. 1987;22(88):827-40.

**Population (n=60)**

1. Aho AL, Paavilainen E, Kaunonen M. Mothers' experiences of peer support via an Internet discussion forum after the death of a child. Scandinavian Journal of Caring Sciences. 2012;26(3):417-26.

2. Baggett KM, Davis B, Feil EG, Sheeber LL, Landry SH, Carta JJ, et al. Technologies for expanding the reach of evidence-based interventions: preliminary results for promoting social-emotional development in early childhood. Topics in Early Childhood Special Education. 2010;29(4):226-38.

3. Barnett B, Corkum P, Elik N. A web-based intervention for elementary school teachers of students with attention-deficit/hyperactivity disorder (ADHD). Psychological Services. 2012;9(2):227-30.

4. Bers MU. New Media for New Organs: A Virtual Community for Pediatric Post-Transplant Patients. Convergence: The Journal of Research into New Media Technologies. 2009;15(4):462-9.

5. Buchanan H, Coulson NS. Accessing dental anxiety online support groups: an exploratory qualitative study of motives and experiences. Patient Education & Counseling. 2007;66(3):263-9.

6. Bull SS, Levine DK, Black SR, Schmiege SJ, Santelli J. Social media-delivered sexual health intervention: a cluster randomized controlled trial. American Journal of Preventive Medicine. 2012;43(5):467-74.

7. Chapple A, Ziebland S. Health Professionals' Attitudes Towards Using a Web 2.0 Portal for Child and Adolescent Diabetes Care: Qualitative Study

2011. p. 173-87.

8. Chen H-H, Yeh M-L, Chao Y-H. Comparing effects of auricular acupressure with and without an internet-assisted program on smoking cessation and self-efficacy of adolescents. Journal of Alternative & Complementary Medicine. 2006;12(2):147-52.

9. Chen HH, Yeh ML. Developing and evaluating a smoking cessation program combined with an Internet-assisted instruction program for adolescents with smoking. Patient Education & Counseling. 2006;61(3):411-8.

10. Clarke JN, Lang L. Mothers whose children have ADD/ADHD discuss their children's medication use: an investigation of blogs. Social Work in Health Care. 2012;51(5):402-16.

11. Coulson NS, Buchanan H. Self-reported efficacy of an online dental anxiety support group: a pilot study. Community Dentistry & Oral Epidemiology. 2008;36(1):43-6.

12. Cunningham JA, van Mierlo T, Fournier R. An online support group for problem drinkers: AlcoholHelpCenter.net. 2008. p. 193-8.

13. Davis JL. Narrative Construction of a Ruptured Self: Stories of Transability on Transabled.org Sociological Perspectives. 2012;55(2):319-40.

14. Dunlop SM, More E, Romer D. Where do youth learn about suicides on the Internet, and what influence does this have on suicidal ideation? Journal of Child Psychology & Psychiatry & Allied Disciplines. 2011;52(10):1073-80.

15. Edward KL, Robins A. Dual diagnosis, as described by those who experience the disorder: Using the Internet as a source of data. International Journal of Mental Health Nursing. 2012;21(6):550-9.

16. Elkin L, Thomson G, Wilson N. Connecting world youth with tobacco brands: YouTube and the internet policy vacuum on Web 2.0. Tobacco Control. 2010;19(5):361-6.

17. Engqvist I, Ferszt G, Ahlin A, Nilsson K. Women's experience of postpartum psychotic episodes--analyses of narratives from the internet. Archives of Psychiatric Nursing. 2011;25(5):376-87.

18. Engqvist I, Nilsson K. Men's experience of their partners' postpartum psychiatric disorders: narratives from the internet. Mental Health in Family Medicine. 2011;8(3):137-46.

19. Evans M, Donelle L, Hume-Loveland L. Social support and online postpartum depression discussion groups: a content analysis. Patient Education & Counseling. 2012;87(3):405-10.

20. Feigelman W, Gorman BS, Beal KC, Jordan JR. Internet support groups for suicide survivors: a new mode for gaining bereavement assistance. Omega - Journal of Death & Dying. 2008;57(3):217-43.

21. Fleischmann A. The hero's story and autism - Grounded theory study of websites for parents of children with autism. Autism. 2005;9(3):299-316.

22. Furmark T, Carlbring P, Hedman E, Sonnenstein A, Clevberger P, Bohman B, et al. Guided and unguided self-help for social anxiety disorder: randomised controlled trial. British Journal of Psychiatry. 2009;195(5):440-7.

23. Gajaria A, Yeung E, Goodale T, Charach A. Beliefs about attention-deficit/hyperactivity disorder and response to stereotypes: youth postings in Facebook groups. Journal of Adolescent Health. 2011;49(1):15-20.

24. Graham AL, Papandonatos GD, Kang H, Moreno JL, Abrams DB. Development and validation of the online social support for smokers scale. Journal of Medical Internet Research. 2011;13(3):e69.

25. Green-Hamann S, Campbell Eichhorn K, Sherblom JC. An Exploration of Why People Participate in Second Life Social Support Groups. Journal of Computer-Mediated Communication. 2011;16(4):465-91.

26. Harvey K, Brown B. Health Communication and Psychological Distress: Exploring the Language of Self-Harm. Canadian Modern Language Review. 2012;68(3):316-40.

27. Hastings SO, Musambira GW, Hoover JD. Community as a key to healing after the death of a child. Communication & Medicine (De Gruyter). 2007;4(2):153-63.

28. Hinduja S, Patchin JW. Bullying, cyberbullying, and suicide. Archives of Suicide Research. 2010;14(3):206-21.

29. Houston TK, Cooper LA, Ford DE. Internet support groups for depression: a 1-year prospective cohort study. American Journal of Psychiatry. 2002;159(12):2062-8.

30. Hubin-Gayte M. Eating disorders and motherhood: Analysis of forums on Internet. Annales Medico-Psychologiques. 2011;169(10):615-20.

31. Johansson R, Sjoberg E, Sjogren M, Johnsson E, Carlbring P, Andersson T, et al. Tailored vs. standardized internet-based cognitive behavior therapy for depression and comorbid symptoms: a randomized controlled trial. PLoS ONE [Electronic Resource]. 2012;7(5):e36905.

32. Jones A, Meier A. Growing [www.parentsofsuicide:](http://www.parentsofsuicide:) A case study of an online support community. Social Work with Groups: A Journal of Community and Clinical Practice. 2011;34(2):101-20.

33. Jones K, Baldwin KA, Lewis PR. The potential influence of a social media intervention on risky sexual behavior and Chlamydia incidence. Journal of Community Health Nursing. 2012;29(2):106-20.

34. Kummervold PE, Gammon D, Bergvik S, Johnsen J-AK, Hasvold T, Rosenvinge JH. Social support in a wired world: use of online mental health forums in Norway. Nordic Journal of Psychiatry. 2002;56(1):59-65.

35. Margalit M, Raskind MH. Mothers of Children with LD and ADHD: Empowerment through Online Communication. Journal of Special Education Technology. 2008;24(1):2008-9.

36. Margalit M, Raskind MH, Higgins EL, Russo-Netzer P. Mothers' Voices on the Internet: Stress, Support and Perceptions of Mothers of Children with Learning Disabilities and Attention Deficit/Hyperactivity Disorder. Learning Disabilities: A Multidisciplinary Journal. 2010;16(1):3-14.

37. Meredith SE, Grabinski MJ, Dallery J. Internet-based group contingency management to promote abstinence from cigarette smoking: A feasibility study. Drug & Alcohol Dependence. 2011;118(1):23-30.

38. Munoz RF, Barrera AZ, Delucchi K, Penilla C, Torres LD, Perez-Stable EJ. International Spanish/English Internet smoking cessation trial yields 20% abstinence rates at 1 year. Nicotine & Tobacco Research. 2009;11(9):1025-34.

39. Ost J, Wright DB, Easton S, Hope L, French CC. Recovered memories, satanic abuse, dissociative identity disorder and false memories in the UK: A survey of clinical psychologists and hypnotherapists. Psychology, Crime & Law. 2013;19(1):1-19.

40. Perini S, Titov N, Andrews G. Clinician-assisted Internet-based treatment is effective for depression: randomized controlled trial. Australian & New Zealand Journal of Psychiatry. 2009;43(6):571-8.

41. Perry-MacLean S-A. Developing interprofessional collaboration using web-based instruction: A case study of the healthy minds/healthy children project. Dissertation Abstracts International Section A: Humanities and Social Sciences. 2011;71(8-A):2753.

42. Putnam JM. Combining Telehealth and E-Learning: a case study in smoking cessation programming American Journal of Health Studies. 2007;22(3):130-8.

43. Rains SA, Keating DM. The Social Dimension of Blogging about Health: Health Blogging, Social Support, and Well-being. Communication Monographs. 2011;78(4):511-34.

44. Robertson L, Skegg K, Poore M, Williams S, Taylor B. An adolescent suicide cluster and the possible role of electronic communication technology. Crisis: Journal of Crisis Intervention & Suicide. 2012;33(4):239-45.

45. Rohrbach LA, Gunning M, Sun P, Sussman S. The Project Towards No Drug Abuse (TND) dissemination trial: implementation fidelity and immediate outcomes.[Erratum appears in Prev Sci. 2010 Mar;11(1):113]. Prevention Science. 2010;11(1):77-88.

46. Sanford AA. 'I Can Air My Feelings Instead of Eating Them': Blogging as Social Support for the Morbidly Obese. Communication Studies. 2010;61(5):567-84.

47. Scharer K. An Internet discussion board for parents of mentally ill young children. Journal of Child & Adolescent Psychiatric Nursing. 2005;18(1):17-25.

48. Sheeber LB, Seeley JR, Feil EG, Davis B, Sorensen E, Kosty DB, et al. Development and pilot evaluation of an Internet-facilitated cognitive-behavioral intervention for maternal depression. Journal of Consulting & Clinical Psychology. 2012;80(5):739-49.

49. Smith DJ, Griffiths E, Poole R, di Florio A, Barnes E, Kelly MJ, et al. Beating Bipolar: exploratory trial of a novel Internet-based psychoeducational treatment for bipolar disorder. Bipolar Disorders. 2011;13(5-6):571-7.

50. Song H, Nam Y, Gould J, Sanders W, McLaughlin M, Fulk J, et al. Cancer survivor identity shared in a social media intervention. Journal of Pediatric Oncology Nursing. 2012;29(2):80-91.

51. Spence J, Titov N, Dear BF, Johnston L, Solley K, Lorian C, et al. Randomized controlled trial of Internet-delivered cognitive behavioral therapy for posttraumatic stress disorder. Depression & Anxiety. 2011;28(7):541-50.

52. Stjernswärd S, Östman M. Illuminating User Experience of a Website for the Relatives of Persons With Depression. International Journal of Social Psychiatry. 2011;57(4):375-86.

53. Takahashi Y, Uchida C, Miyaki K, Sakai M, Shimbo T, Nakayama T. Potential benefits and harms of a peer support social network service on the internet for people with depressive tendencies: qualitative content analysis and social network analysis. Journal of Medical Internet Research. 2009;11(3):e29.

54. Terbeck S, Chesterman LP. Parents, ADHD and the internet. Attention Deficit and Hyperactivity Disorders. 2012;4(3):159-66.

55. Thompson R. Screwed up, but working on it: (Dis)ordering the self through e-stories. Narrative Inquiry. 2012;22(1):86-104.

56. Titov N, Andrews G, Choi I, Schwencke G, Mahoney A. Shyness 3: randomized controlled trial of guided versus unguided Internet-based CBT for social phobia. Australian & New Zealand Journal of Psychiatry. 2008;42(12):1030-40.

57. Titov N, Andrews G, Johnston L, Robinson E, Spence J. Transdiagnostic Internet treatment for anxiety disorders: A randomized controlled trial. Behaviour Research & Therapy. 2010;48(9):890-9.

58. Titov N, Dear BF, Schwencke G, Andrews G, Johnston L, Craske MG, et al. Transdiagnostic internet treatment for anxiety and depression: a randomised controlled trial. Behaviour Research & Therapy. 2011;49(8):441-52.

59. Trondsen MV. Living with a mentally ill parent: exploring adolescents' experiences and perspectives. Qualitative Health Research. 2012;22(2):174-88.

60. van der Houwen K, Stroebe M, Schut H, Stroebe W, van den Bout J. Online mutual support in bereavement: An empirical examination. Computers in Human Behavior. 2010;26(6):1519-25.

**Topic (n=77)**

1. Abascal L, Bruning Brown J, Winzelberg AJ, Dev P, Taylor CB. Combining universal and targeted prevention for school-based eating disorder programs. International Journal of Eating Disorders. 2004;35(1):1-9.

2. Aoyama I, Saxon TF, Fearon DD. Internalizing Problems among Cyberbullying Victims and Moderator Effects of Friendship Quality. Multicultural Education and Technology Journal. 2011;5(2):92-105.

3. Ashurst EJ, Jones RB, Williamson GR, Emmens T, Perry J. Collaborative learning about e-health for mental health professionals and service users in a structured anonymous online short course: pilot study. BMC Medical Education. 2012;12:37.

4. Bardone-Cone AM, Cass KM. What does viewing a pro-anorexia website do? An experimental examination of website exposure and moderating effects. International Journal of Eating Disorders. 2007;40(6):537-48.

5. Bardone-Cone AMC, Kamila M. Investigating the impact of pro-anorexia websites: a pilot study. European Eating Disorders Review. 2006;14(4):256-62.

6. Bennett DC, Guran EL, Ramos MC, Margolin G. College students' electronic victimization in friendships and dating relationships: anticipated distress and associations with risky behaviors. Violence & Victims. 2011;26(4):410-29.

7. Borzekowski DLG, Schenk S, Wilson JL, Peebles R. e-Ana and e-Mia: A content analysis of pro-eating disorder Web sites. American Journal of Public Health. 2010;100(8):1526-34.

8. Brown JB, Winzelberg AJ, Abascal LB, Taylor CB. An evaluation of an Internet-delivered eating disorder program for adolescents and their parents [corrected] [published erratum appears in Public Health Rep 2010 Nov-Dec;125(6):788]. Journal of Adolescent Health. 2004;35(4):290-6.

9. Bruning Brown J, Winzelberg AJ, Abascal LB, Taylor CB. An evaluation of an Internet-delivered eating disorder prevention program for adolescents and their parents. Journal of Adolescent Health. 2004;35(4):290-6.

10. Bruning Brown JL. An evaluation of an Internet-delivered eating disorder prevention program for adolescents and their parents. Dissertation Abstracts International: Section B: The Sciences and Engineering. 2003;63(10-B):4891.

11. Burns JM, Durkin LA, Nicholas J. Mental health of young people in the United States: what role can the internet play in reducing stigma and promoting help seeking? Journal of Adolescent Health. 2009;45(1):95-7.

12. Castro TS, Osório A. Online violence: Not beautiful enough... not thin enough. Anorectic testimonials in the web. PsychNology Journal. 2012;10(3):169-86.

13. Celio AA, Winzelberg AJ, Wilfley DE, Eppstein-Herald D, Springer EA, Dev P, et al. Reducing risk factors for eating disorders: Comparison of an Internet- and a classroom-delivered psychoeducational program. Journal of consulting and clinical psychology. 2000;68(4):650-7.

14. Celio AA. Early intervention of eating- and weight-related problems via the internet in overweight adolescents: A randomized controlled trial. Dissertation Abstracts International: Section B: The Sciences and Engineering. 2005;66(4-B):2299.

15. Clarke JN, Sargent C. Childhood depression: Parents talk with one another on the 'net. Social Work in Mental Health. 2010;8(6):510-25.

16. Csipke E, Horne O. Pro-eating disorder websites: users' opinions. European Eating Disorders Review. 2007;15(3):196-206.

17. Demaso DR, Marcus NE, Kinnamon C, Gonzalez-Heydrich J. Depression experience journal: a computer-based intervention for families facing childhood depression. Journal of the American Academy of Child & Adolescent Psychiatry. 2006;45(2):158-65.

18. Dempsey AG, Sulkowski ML, Nichols R, Storch EA. Differences between Peer Victimization in Cyber and Physical Settings and Associated Psychosocial Adjustment in Early Adolescence. Psychology in the Schools. 2009;46(10):962-72.

19. Egan KG, Koff RN, Moreno MA. College Students' Responses to Mental Health Status Updates on Facebook. Issues in Mental Health Nursing. 2013;34(1):46-51.

20. Egan KGM, Megan A. Prevalence of stress references on college freshmen Facebook profiles. CIN: Computers, Informatics, Nursing. 2011;29(10):586-92.

21. Eichhorn KC. Soliciting and Providing Social Support Over the Internet: An Investigation of Online Eating Disorder Support Groups. Kontaktanbahnung und soziale Unterstützung mit Hilfe des Internets: Eine Untersuchung von Online-Selbsthilfegruppen zum Thema Essstörungen. 2008;14(1):67-78.

22. Ellis LA, Campbell AJ, Sethi S, O'Dea BM. Comparative randomized trial of an online cognitive-behavioral therapy program and an online support group for depression and anxiety. Journal of CyberTherapy and Rehabilitation. 2011;4(4):461-7.

23. Fichter MM, Quadflieg N, Nisslmuller K, Lindner S, Osen B, Huber T, et al. Does internet-based prevention reduce the risk of relapse for anorexia nervosa? Behaviour Research & Therapy. 2012;50(3):180-90.

24. Fox N, Ward K, O'Rourke A. Pro-anorexia, weight-loss drugs and the internet: an "anti-recovery" explanatory model of anorexia. Sociology of Health & Illness. 2005;27(7):944-71.

25. Freeman E, Barker C, Pistrang N. Outcome of an online mutual support group for college students with psychological problems. Cyberpsychology & Behavior. 2008;11(5):591-3.

26. Goebert D, Else I, Matsu C, Chung-Do J, Chang JY. The impact of cyberbullying on substance use and mental health in a multiethnic sample. Maternal & Child Health Journal. 2011;15(8):1282-6.

27. Greidanus E. Online help seeking. Dissertation Abstracts International: Section B: The Sciences and Engineering. 2011;71(12-B):7755.

28. Haas SM, Irr ME, Jennings NA, Wagner LM. Communicating thin: A grounded model of Online Negative Enabling Support Groups in the pro-anorexia movement. New Media & Society. 2011;13(1):40-57.

29. Habermeyer E, Habermeyer V, Jahn K, Domes G, Nagel E, Herpertz SC. [An internet based discussion board for persons with borderline personality disorders moderated health care professionals]. Psychiatrische Praxis. 2009;36(1):23-9.

30. Hawdon J, Ryan J. Well-being after the Virginia Tech mass murder: The relative effectiveness of face-to-face and virtual interactions in providing support to survivors. Traumatology. 2012;18(4):3-12.

31. Hinduja S, Patchin JW. Offline consequences of online victimization: school violence and delinquency. Journal of School Violence. 2007;6(3):89-112.

32. Jacobi C, Morris L, Beckers C, Bronisch-Holtze J, Winter J, Winzelberg AJ, et al. Maintenance of internet-based prevention: a randomized controlled trial. International Journal of Eating Disorders. 2007;40(2):114-9.

33. Jacobi C, Volker U, Trockel MT, Taylor CB. Effects of an Internet-based intervention for subthreshold eating disorders: a randomized controlled trial. Behaviour Research & Therapy. 2012;50(2):93-9.

34. Jett S, LaPorte DJ, Wanchisn J. Impact of exposure to pro-eating disorder websites on eating behaviour in college women. European Eating Disorders Review. 2010;18(5):410-6.

35. Jones M, Luce KH, Osborne MI, Taylor K, Cunning D, Doyle AC, et al. Randomized, controlled trial of an internet-facilitated intervention for reducing binge eating and overweight in adolescents. Pediatrics. 2008;121(3):453-62.

36. Jones M. Reducing binge eating and overweight in adolescents via the Internet. Dissertation Abstracts International: Section B: The Sciences and Engineering. 2010;71(3-B):2050.

37. Jones M, Volker U, Lock J, Taylor CB, Jacobi C. Family-based early intervention for anorexia nervosa. European Eating Disorders Review. 2012;20(3):e137-43.

38. Juarascio AS, Shoaib A, Timko CA. Pro-eating disorder communities on social networking sites: a content analysis. Brunner-Mazel Eating Disorders Monograph Series. 2010;18(5):393-407.

39. Keski-Rahkonen A, Tozzi F. The process of recovery in eating disorder sufferers' own words: an Internet-based study. International Journal of Eating Disorders. 2005;37 Suppl:S80-6; discussion S7-9.

40. Lapinski MK. StarvingforPerfect.com: A Theoretically Based Content Analysis of Pro-Eating Disorder Web Sites. Health Communication. 2006;20(3):243-53.

41. Lyons EJ, Mehl MR, Pennebaker JW. Pro-anorexics and recovering anorexics differ in their linguistic Internet self-presentation. Journal of Psychosomatic Research. 2006;60(3):253-6.

42. Machmutow K, Perren S, Sticca F, Alsaker FD. Peer Victimisation and Depressive Symptoms: Can Specific Coping Strategies Buffer the Negative Impact of Cybervictimisation? Emotional and Behavioural Difficulties. 2012;17(3):403-20.

43. Maloney P. Online networks and emotional energy. Information, Communication & Society. 2013;16(1):105-24.

44. Manwaring JL, Bryson SW, Goldschmidt AB, Winzelberg AJ, Luce KH, Cunning D, et al. Do adherence variables predict outcome in an online program for the prevention of eating disorders? Journal of Consulting & Clinical Psychology. 2008;76(2):341-6.

45. Marcus MA, Westra HA, Eastwood JD, Barnes KL, Mobilizing Minds Research G. What are young adults saying about mental health? An analysis of Internet blogs. Journal of Medical Internet Research. 2012;14(1):e17.

46. Martin EA, Bailey DH, Cicero DC, Kerns JG. Social networking profile correlates of schizotypy. Psychiatry Research. 2012;200(2-3):641-6.

47. McCabe J. Resisting Alienation: The Social Construction of Internet Communities Supporting Eating Disorders. Communication Studies. 2009;60(1):1-16.

48. Miller JD, Hufstedler SM. Cyberbullying Knows No Borders. Australian Teacher Education Association Paper presented at the Annual Conference of the Australian Teacher Education Association. 2009.

49. Moreno MA, Jelenchick LA, Egan KG, Cox E, Young H, Gannon KE, et al. Feeling bad on Facebook: depression disclosures by college students on a social networking site. Depression & Anxiety. 2011;28(6):447-55.

50. Moreno MA, Christakis DA, Egan KG, Brockman LN, Becker T. Associations between displayed alcohol references on Facebook and problem drinking among college students. Archives of Pediatrics & Adolescent Medicine. 2012;166(2):157-63.

51. Mulveen R, Hepworth J. An interpretative phenomenological analysis of participation in a pro-anorexia internet site and its relationship with disordered eating. Journal of Health Psychology. 2006;11(2):283-96.

52. Nedoschill J, Leiberich P, Popp C, Loew T. [[www.hungrig-online.de:](http://www.hungrig-online.de:) results from an online survey in the largest German-speaking Internet self help community for eating disorders]. Praxis der Kinderpsychologie und Kinderpsychiatrie. 2005;54(9):728-41.

53. Peebles R, Harrison S, McCown K, Wilson J, Borzekowski D, Lock J. 101. Voices of Pro-Ana and Pro-Mia: A Qualitative Analysis of Reasons for Entering and Continuing Pro-Eating Disorder Website Usage. Journal of Adolescent Health. 2012;50(2):S62.

54. Perren S, Dooley J, Shaw T, Cross D. Bullying in school and cyberspace: Associations with depressive symptoms in Swiss and Australian adolescents. Child and Adolescent Psychiatry and Mental Health. 2010;4:28.

55. Pierre-Gerard S, Perret-Catipovic M. When adolescents tell us about suicide on the Internet, how do they say it to us? Neuropsychiatrie de l'Enfance et de l'Adolescence. 2009;57(2):128-35.

56. Pretorius N, Arcelus J, Beecham J, Dawson H, Doherty F, Eisler I, et al. Cognitive-behavioural therapy for adolescents with bulimic symptomatology: the acceptability and effectiveness of internet-based delivery. Behaviour Research & Therapy. 2009;47(9):729-36.

57. Pretorius N, Rowlands L, Ringwood S, Schmidt U. Young people's perceptions of and reasons for accessing a web-based cognitive behavioural intervention for bulimia nervosa. European Eating Disorders Review. 2010;18(3):197-206.

58. Pryslopski HA. Reality TV programs, Internet use, and eating disorder symptoms. Dissertation Abstracts International: Section B: The Sciences and Engineering. 2012;72(12-B):7695.

59. Ransom DC, La Guardia JG, Woody EZ, Boyd JL. Interpersonal interactions on online forums addressing eating concerns. International Journal of Eating Disorders. 2010;43(2):161-70.

60. Ridout B, Campbell A, Ellis L. 'Off your Face(book)': alcohol in online social identity construction and its relation to problem drinking in university students. Drug & Alcohol Review. 2012;31(1):20-6.

61. Rodgers RF, Skowron S, Chabrol H. Disordered eating and group membership among members of a pro-anorexic online community. European Eating Disorders Review. 2012;20(1):9-12.

62. Santor DA, Poulin C, LeBlanc JC, Kusumakar V. Online health promotion, early identification of difficulties, and help seeking in young people. Journal of the American Academy of Child & Adolescent Psychiatry. 2007;46(1):50-9.

63. Schroeder PA. Adolescent girls in recovery for eating disorders: Exploring past pro-anorexia internet community experiences. Dissertation Abstracts International: Section B: The Sciences and Engineering. 2010;71(2-B):1354.

64. Siriaraya P, Tang C, Ang CS, Pfeil U, Zaphiris P. A Comparison of Empathic Communication Pattern for Teenagers and Older People in Online Support Communities. Behaviour and Information Technology. 2011;30(5):617-28.

65. Sourander A, Brunstein Klomek A, Ikonen M, Lindroos J, Luntamo T, Koskelainen M, et al. Psychosocial risk factors associated with cyberbullying among adolescents: a population-based study. Archives of General Psychiatry. 2010;67(7):720-8.

66. Taylor CB, Bryson S, Luce KH, Cunning D, Doyle AC, Abascal LB, et al. Prevention of eating disorders in at-risk college-age women. Archives of General Psychiatry. 2006;63(8):881-8.

67. Ubertini M. Cyberbullying May Reduce Adolescent's Well-Being: Can Life Satisfaction and Social Support Protect Them? ProQuest LLC PsyD Dissertation, Hofstra University. 2010.

68. Vicary AM, Fraley RC. Student reactions to the shootings at Virginia Tech and Northern Illinois University: Does sharing grief and support over the internet affect recovery? Personality & Social Psychology Bulletin. 2010;36(11):1555-63.

69. Volker UJ, C.;Barr Taylor, C. Adaptation and evaluation of an Internet-based prevention program for eating disorders in a sample of women with subclinical eating disorder symptoms: a pilot study. Eating & Weight Disorders: EWD. 2011;16(4):e270-3.

70. Völlink T, Bolman CA, Dehue F, Jacobs NC. Coping with Cyberbullying: Differences Between ictims, Bully-victims and Children not Involved in Bullying. Journal of Community & Applied Social Psychology. 2013;23(1):7-24.

71. Williams S, Reid M. Understanding the experience of ambivalence in anorexia nervosa: the maintainer's perspective. Psychology & Health. 2010;25(5):551-67.

72. Wilson JL, Peebles R, Hardy KK, Litt IF. Surfing for thinness: a pilot study of pro-eating disorder Web site usage in adolescents with eating disorders. Pediatrics. 2006;118(6):e1635-43.

73. Winzelberg AJ, Taylor CB, Sharpe T, Eldredge KL, Dev P, Constantinou PS. Evaluation of a computer-mediated eating disorder intervention program. International Journal of Eating Disorders. 1998;24(4):339-49.

74. Winzelberg AJ, Eppstein D, Eldredge KL, Wilfley D, Dasmahapatra R, Dev P, et al. Effectiveness of an Internet-based program for reducing risk factors for eating disorders. Journal of Consulting & Clinical Psychology. 2000;68(2):346-50.

75. Ybarra MLM, K. J. Prevalence and frequency of Internet harassment instigation: implications for adolescent health. Journal of Adolescent Health. 2007;41(2):189-95.

76. Zabinski MF, Pung MA, Wilfley DE, Eppstein DL, Winzelberg AJ, Celio A, et al. Reducing risk factors for eating disorders: targeting at-risk women with a computerized psychoeducational program. International Journal of Eating Disorders. 2001;29(4):401-8.

77. Zaluska M, Bronowski P, Panasiuk K, Brykalski J, Paszko J. [Evaluation of the ICAR program--Internet communication and active rehabilitation for people with mental disorders]. Psychiatria Polska. 2008;42(3):353-64.

**Non-English (n=11)**

1. Abajo E. A Web nursing intervention to prevent eating disorders [Spanish]. Nure Investigación. 2008(32):24p.

2. Ambrosi-Randic N, Pokrajac-Bulian A, Ogresta J, Lacovich M. Eating disorders and internet: Content analysis of Croatian web pages. Psihologijske Teme. 2008;17(1):37-55.

3. Cerna A, Smahel D. Self-injury in adolescence: Blog as a mean of community formation. Ceskoslovenska Psychologie. 2009;53(5):492-504.

4. De Rosa C, Del Vecchio V, Del Gaudio L, Sampogna G, Luciano M, Giacco D, et al. Suicide and the Internet: A search on Italian websites. Giornale Italiano di Psicopatologia / Italian Journal of Psychopathology. 2011;17(4):376-82.

5. Eichenberg C, Flumann A, Hensges K. Pro-ana communities on the Internet. Survey of users. Psychotherapeut. 2011;56(6):492-500.

6. Estevez A, Villardon L, Calvete E, Padilla P, Orue I. Adolescent victims of cyberbullying: Prevalence and characteristics. Behavioral Psychology / Psicologia Conductual: Revista Internacional Clinica y de la Salud. 2010;18(1):73-89.

7. Fernandez-Davila P, Lupianez-Villanueva F, Zaragoza Lorca K. [Attitudes toward online HIV/sexually-transmitted infection prevention programs and Internet user profiles among men who have sex with men]. Gaceta Sanitaria. 2012;26(2):123-30.

8. Mokkenstorm J, Huisman A, Kerkhof A. [Suicide prevention via the internet and the telephone: 113Online]. Tijdschrift voor Psychiatrie. 2012;54(4):341-8.

9. Theis F, Wolf M, Fiedler P, Backenstrass M, Kordy H. [Eating disorders on the internet: an experimental study on the effects of pro-eating disorders websites and self-help websites]. Psychotherapie, Psychosomatik, Medizinische Psychologie. 2012;62(2):58-65.

10. Vagnhammar SR. [Suicide sites on the Internet--an alarming phenomenon]. Lakartidningen. 2004;101(49):4028, 31-2.

11. Winkel S, Groen G, Petermann F. [Social support in suicide forums]. Praxis der Kinderpsychologie und Kinderpsychiatrie. 2005;54(9):714-27.

**Duplicate (n=10)**

1. Ambrosi-Randić NP-B, Alessandra;Ogresta, Jelena;Lacovich, Mauro. Eating Disorders and Internet: Content Analysis of Croatian Web Pages. Psihologijske Teme. 2008;17(1):37-55.

2. Cox M, Scharer K, Clark A. Development of a Web-based program to improve communication about sex. CIN: Computers, Informatics, Nursing. 2009;27(1):18-25.

3. Doyle A, Goldschmidt A, Huang C, Winzelberg A, Taylor C, Wilfley DE. Reduction of overweight and eating disorder symptoms via the Internet in adolescents: a randomized controlled trial. Journal of Adolescent Health. 2008;43(2):172-9.

4. Gow R, Trace S, Mazzeo S. Preventing weight gain in first year college students: an online intervention to prevent the "freshman fifteen". Eating Behaviors. 2010;11(1):33-9.

5. Greidanus E, Everall R. Helper Therapy in an Online Suicide Prevention Community. British Journal of Guidance and Counselling. 2010;38(2):191-204.

6. Linkletter M, Gordon K, Dooley J. The choking game and YouTube: a dangerous combination. Clinical Pediatrics. 2010;49(3):274-9.

7. Nordqvist C, Hanberger L, Timpka T, Nordfeldt S. Health Professionals' Attitudes Towards Using a Web 2.0 Portal for Child and Adolescent Diabetes Care: Qualitative Study. 2009. p. 1.

8. Osvaldsson K. Bullying in Context: Stories of Bullying on an Internet Discussion Board. Children and Society. 2011;25(4):317-27.

9. Rydell S, French S, Fulkerson J, Neumark-Sztainer D, Gerlach AF, Story M, et al. Use of a Web-based component of a nutrition and physical activity behavioral intervention with Girl Scouts. Journal of the American Dietetic Association. 2005;105(9):1447-50.

10. Sharkey S, Smithson J, Hewis E, Jones R, Emmens T, Ford T, et al. Supportive interchanges and face-work as 'protective talk' in an online self-harm support forum. Communication & Medicine 2012;9(1):71-82.
