# Supplementary material for: A Systematic Review of Social Media Use to Discuss and View Deliberate Self-Harm Acts
Source: PLoS One. 2016 May 18;11(5):e0155813. doi: 10.1371/journal.pone.0155813 (PMC4871432; doi:10.1371/journal.pone.0155813)
Supplement: S4 Appendix — (DOCX) [file pone.0155813.s004.docx]

**S4 Appendix. Mixed Methods Appraisal Tool Scoring of Included Studies (n=26)^[[1]](#footnote-1)^**

| **Study** | **Primary Study Design** | **Methodological quality criteria*** | | | | **Overall Score** |
| --- | --- | --- | --- | --- | --- | --- |
|  | *Qualitative* | 1.1 Relevant data sources | 1.2 Relevant data analysis | 1.3 Appropriate consideration of how findings relate to context | 1.4 Appropriate consideration of how findings relate to researchers’ influence |  |
| Cash, 2013^19^ |  | Yes | Yes | Yes | Yes | 100% |
| Rodham, 2007^20^ |  | Yes | Yes | Yes | Yes | 100% |
| Lewis, 2012^21^ |  | Yes | Yes | Yes | Yes | 100% |
| Niwa, 2012^33^ |  | Yes | Yes | Yes | Yes | 100% |
| Baker, 2008^34^ |  | Yes | Yes | Yes | Yes | 100% |
| Miller, 1998^35^ |  | Yes | Yes | Yes | Yes | 100% |
| Smithson, 2011^36^ |  | Yes | Yes | Yes | Yes | 100% |
| Horne, 2009^37^ |  | Yes | Yes | Yes | No | 75% |
| Smithson, 2011^38^ |  | Yes | Yes | Yes | Yes | 100% |
| Adler, 2008^39^ |  | Yes | Yes | Yes | No | 75% |
| Sharkey, 2012^40^ |  | Can’t Tell | Yes | Yes | Yes | 75% |
| Greidanus, 2010^44^ |  | Yes | Yes | Yes | Can’t Tell | 75% |
| Fekete, 2002^46^ |  | Yes | Yes | Yes | Yes | 100% |
| Gradin, 2011^47^ |  | Can’t Tell | Yes | Yes | Can’t Tell | 50% |
| Whitlock, 2006^49^** |  | Yes | Yes | Yes | Yes | 50% |
|  | *Quantitative Non-Randomized* | 3.1 Recruitment minimizes selection bias | 3.2 Appropriate measurements of exposure/intervention and outcomes | 3.3 Comparable participants between groups | 3.4 Complete outcome data/acceptable response rate/acceptable follow-up |  |
| Masuda, 2013^28^ |  | Yes | Yes | Yes | Yes | 100% |
|  | *Quantitative Descriptive* | 4.1 Relevant sampling strategy | 4.2 Representative sample | 4.3 Appropriate measurements | 4.4 Acceptable response rate |  |
| Lewis, 2014^18^ |  | Yes | Yes | Yes | Can’t Tell | 75% |
| Sueki, 2012^29^ |  | Can’t Tell | Can’t Tell | Can’t Tell | Yes | 25% |
| Eichenberg, 2006^30^ |  | Yes | Can’t Tell | Can’t Tell | Can’t Tell | 25% |
| Murray, 2006^31^ |  | Yes | Can’t Tell | Can’t Tell | Can’t Tell | 25% |
| Jones, 2011^32^ |  | Yes | Can’t Tell | Can’t Tell | Yes | 50% |
| Linkletter, 2010^41^ |  | Yes | Can’t Tell | Yes | Can’t Tell | 50% |
| Lewis, 2011^42^ |  | Can’t Tell | Can’t Tell | Yes | Can’t Tell | 25% |
| Barak, 2006^43^ |  | Yes | Can’t Tell | Yes | Can’t Tell | 50% |
| Duggan, 2012^45^ |  | Yes | Yes | Yes | Can’t Tell | 75% |
| Lewis, 2011^47^ |  | Yes | Yes | Yes | Can’t Tell | 75% |
| Whitlock, 2006^49^** |  | Yes | Can’t Tell | Yes | Can’t Tell | 50% |
|  | *Mixed Methods* | 5.1 Relevant research design | 5.2 Relevant integration of qualitative and quantitative data | 5.3 Appropriate consideration of limitations of data integration |  |  |
| Whitlock, 2006^49^** |  | Yes | Yes | Can’t Tell |  | 50% |

* All included studies met the criteria for the screening questions; therefore these are not included in the table. No included studies were assessed as quantitative randomized controlled trials, and this category of the Mixed Methods Appraisal Tool has been omitted from the table.

** Mixed methods studies are assessed for the qualitative component, the relevant quantitative component, and the mixed methods component, and therefore appear three times in the table, in each of the applicable sections.

1. Adapted from: Pluye P, Robert E, Cargo M, et al. Proposal: A mixed methods appraisal tool for systematic mixed studies reviews. 2011; <http://mixedmethodsappraisaltoolpublic.pbworks.com>. [↑](#footnote-ref-1)
